# Enzymatically catalyzed furan-based copolyesters containing dilinoleic diol as a building block†

**DOI:** 10.1039/d3ra03885h

**Published:** 2023-07-24

**Authors:** Martyna Sokołowska, Jagoda Nowak-Grzebyta, Ewa Stachowska, Piotr Miądlicki, Magdalena Zdanowicz, Beata Michalkiewicz, Miroslawa El Fray

**Affiliations:** a West Pomeranian University of Technology, Szczecin, Faculty of Chemical Technology and Engineering, Department of Polymer and Biomaterials Science Al. Piastow 45 71-311 Szczecin Poland mirfray@zut.edu.pl; b Poznan University of Technology, Faculty of Mechanical Engineering Ul. Piotrowo 3 60-965 Poznan Poland; c West Pomeranian University of Technology, Szczecin, Faculty of Chemical Technology and Engineering, Engineering of Catalytic and Sorbent Materials Department Al. Piastow 45 71-311 Szczecin Poland; d West Pomeranian University of Technology in Szczecin, Faculty of Food Sciences, Center of Bioimmobilisation and Innovative Packaging Materials Ul. Janickiego 35 71-270 Szczecin Poland

## Abstract

A more environmentally friendly method for creating sustainable alternatives to traditional aromatic–aliphatic polyesters is a valuable step towards resource-efficiency optimization. A library of furan-based block copolymers was synthesized *via* temperature-varied two-step polycondensation reaction in diphenyl ether using *Candida antarctica* lipase B (CAL-B) as a biocatalyst where dimethyl 2,5-furandicarboxylate (DMFDCA), α,ω-aliphatic linear diols (α,ω-ALD), and bio-based dilinoleic diol (DLD) were used as the starting materials. Nuclear magnetic spectroscopy (^1^H and ^13^C NMR), Fourier transform spectroscopy (FTIR) and size exclusion chromatography (SEC) were used to analyze the resulting copolymers. Additionally, crystallization behavior and thermal properties were studied using X-ray diffraction (XRD), digital holographic microscopy (DHM), and differential scanning microscopy (DSC). Finally, oxygen transmission rates (OTR) and dynamic mechanical analysis (DMTA) of furan-based copolyesters indicated their potential for medical packaging.

## Abstract 2

Enzymatically-catalyzed polycondensation as more environmentally friendly method for creating sustainable alternatives to traditional aromatic–aliphatic polyesters is a valuable step towards resource-efficiency optimization.

## Graphical abstract

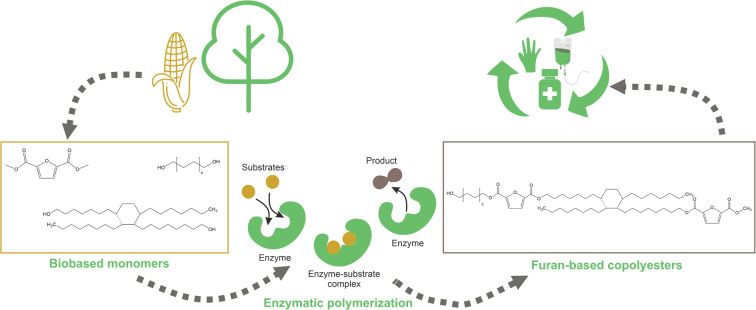

## 1. Introduction

**Fig. 1:**
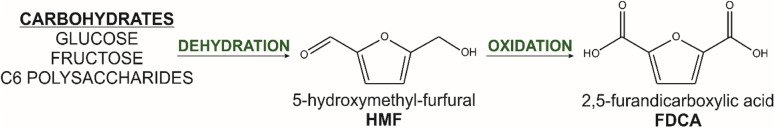
General procedure to produce HMF and FDCA from hexoses and C6 polysaccharides.

Nowadays, cumulative environmental pollution, greenhouse gas emissions, and climate change are leading to the development of new ideas and solutions which need to be implemented to reduce the undesirable effects caused by the processing of fossil resources.^1,2^ Therefore, the use of biomass as a raw material for the production of chemicals has been a focus for many years as concerns over the sustainability of chemical manufacturing have become increasingly important.^3–14^ One particular area of research focus involves the substitution of terephthalic acid (TPA) with 2,5-furandicarboxylic acid (FDCA) due to their similar chemical structures. FDCA presents a compelling alternative as it can be obtained entirely from renewable resources through the oxidative conversion of 5-hydroxymethylfurfural (HMF), which is derived from the acidic dehydrogenation of hexoses (Fig. 1). Hexoses, primarily originating from plant biomass, play a crucial role as monomers and serve as a significant component of the renewable feedstock utilized in FDCA production. Plant-based feedstocks, including agricultural residues, energy crops, and forest residues, serve as valuable sources for hexose production.^3^ These plant materials undergo biomass conversion and biorefining processes to extract and hydrolyze the complex carbohydrates into their constituent hexose monomers. The utilization of FDCA, in conjunction with various monomers, has been extensively explored and documented in numerous published papers and approaches.^4–13^ Notably, significant attention has been dedicated to the development of semicrystalline polyesters and copolyesters. These materials possess several advantageous features and find prevalent applications as engineering, packaging, textile, and elastomeric materials.^14^

One of the materials that present special potential as biobased alternatives for TPA-based polymers such as poly(ethylene terephthalate) (PET) or poly(butylene terephthalate) (PBT) are poly(ethylene furanoate) (PEF) and poly(butylene furanoate) (PBF), whereas PEF is already produced at pilot scale and used in commercial applications as packaging material for soft drinks, films, and fibers.^15^ These semicrystalline engineering thermoplastics exhibit similar and even better mechanical properties, increased chemical and thermal stability, electrical insulation, excellent barrier properties, and higher ability of polymer chain orientation compared to its TPA-based analogs.^16,17^ For example, Papageorgiou *et al.* analyzed in detail the phase transitions and crystal structure of 2,5-FDCA-based and TPA-based polyesters.^11,18^ The obtained results suggest that barrier properties against oxygen, carbon dioxide, and water vapor were significantly improved in the case of 2,5-FDCA analog compared to PET, which means that these materials could be suitable for food packaging applications. Moreover, based on Knoop and co-workers, the mechanical properties of 2,5-FDCA polyesters are comparable to those of PET.^19^ On the other hand, Jiang *et al.* showed that polyesters obtained *via* polycondensation of 2,5-FDCA with ethylene glycol (EG), propylene glycol (PG), and butylene glycol (BG) possess a lower melting temperature, higher glass transition temperature, and slightly lower thermal stability in comparison to TPA-based counterparts.^13^ These results also revealed that all polymers are semicrystalline materials but crystallize slower than polyesters possessing a benzene ring and these features are beneficial from a processing point of view, as the lower melting temperature combined with still high heat resistance facilitates the film blowing and extrusion processes which directly translate to lower energy consumption.

It is undeniable, that above mentioned furan-based polyesters are characterized by outstanding and promising properties, however, their synthesis is usually performed using metal catalysts at a high temperature above 200 °C. At that temperature range, FDCA can undergo decomposition or side reactions which may lead to material discoloration.^20^ Therefore, there is a need to search for a new catalytic system that will enable to perform synthesis in lower temperatures and replace rather toxic tetrabutyl titanate or Sb_3_O_4_ whose residues may remain in the material after synthesis and be harmful to the environment. Thus, to contribute to the principles of sustainable development of polymers by using renewable raw materials, it is necessary to also think about catalysts to create an entirely eco-friendly pathway for furan-based polyester production.

Enzymes are an interesting alternative as Nature's catalysts which has a chance to replace conventional techniques and physical modification methods.^21^ They exhibit a number of advantages, including high efficiency, substrate specificity, catalyst recyclability, and the ability to work under mild reaction conditions.^22^ Lipases are the most widely employed enzymes in organic reactions such as transesterification, esterification, aminolysis, and Michel addition.^23^ Obtained from yeast species: *Candida antarctica* lipase B (CAL-B) is a good catalyst for these reactions because of its stability and high reactivity. Moreover, the unique structure of CAL-B active site pocket is responsible for the high regio-, enantio-, and stereoselectivity of this enzyme due to which it is possible to obtain materials with a well-defined chemical structure which is a desirable feature *e.g.* in the pharmaceutical industry.^24^ We also confirmed this fact in our previous work^26^ where the poly(butylene succinate)-based copolyesters obtained with the use of CAL-B were characterized by a more regular structure compared to the materials synthesized using a metal catalyst.^25^ Notably, besides succinate-based polyesters^26–28^ many types of other polymers have been successfully synthesized *via* CAL-B-catalyzed polymerization, including polyesters such as 2,5-furandicarboxylic acid-based polyesters,^10,12,28^ vegetal oil-based polyesters,^29^ sugar-based polyesters,^30^ and 3,6-dianhydrohexitol-based polyesters.^31^ It is also noteworthy that the immobilized form of CAL-B can be conveniently removed from the reaction mixture through filtration, unlike metal catalysts which are prone to strong metal–metal interactions and often remain in the final product, making removal difficult. This makes enzymatic catalysts a more desirable option for resource-efficient and optimized synthesis processes.

Dimerization of fatty acids mainly C18 vegetable unsaturated fatty acids like oleic or linoleic acid is triggering increasing attention in sustainable polymer chemistry. Subsequent hydrogenation or reduction of C18 fatty acids yields difunctional monomers: fatty dimer diols or fatty dimer acids characterized by low glass transition temperature, amorphous structure, and high conformational flexibility of aliphatic chains. They are attractive biobased components to create soft segments within copolyesters due to the low cohesion energy (2.85 kJ mol^−1^) which gives the ability of free rotation of carbon–carbon bonds (the energy barrier of the rotation of carbon–carbon bonds is 12.6 kJ mol^−1^).^32^ It was already proved in our previous studies where enzymatic synthesis of poly(butylene succinate)-*co*-(dilinoleic succinate) multiblock copolyesters was performed, and where dilinoleic diol (DLD) being a dimerized fatty acid derivative was used as a building block to create soft segments.^25,33^ Notably, study by Kwiatkowska *et al.* demonstrated the synthesis of 2,5-poly(butylene furanoate) catalyzed by organometallic catalyst with variable content of DLD indicating successful direct esterification of FDCA with 1,3-propanediol and subsequent copolymerization with DLD.^7^ Interestingly enough, in recent work, Nasr *et al.* conducted enzymatic polycondensation reactions using diethyl 2,5-furandicarboxylate, 1,4-butanediol, 1,8-octanediol, and Pripol™ 2033 in their work. They referred to Pripol™ 2033 as fatty dimer diol in their study, while in this work, the same compound is referred to as dilinoleic diol (DLD). The study revealed that the addition of Pripol™ 2033 enhanced the polycondensation reaction in systems that contained high furan content and 1,4-butanediol, which otherwise did not produce any high molecular weight products.^34^

The primary objective of this research is to broaden the scope of polyester materials that can be synthesized from renewable resources by employing CAL-B as a biocatalyst and to deliver a comprehensive characterization of novel furan-based block copolyesters. Specifically, the investigation will delve into the influence of diol chain lengths (*C* = 6, 8, 10, 12) in alkyl furanoate hard segments, examining their impact on synthesis efficiency, crystalline structure, and thermal properties of poly(alkyl furanoate-*co*-dilinoleic furanoate) copolyesters with a 70–30 wt% ratio of hard to soft segments. Moreover, the resulting materials underwent thorough assessment for their potential applicability as eco-friendly packaging materials in the medical sector to create “sterile barrier system” (SBS), where allowing sterilization, providing an acceptable microbial barrier and allowing aseptic presentation are of paramount importance.^35,36^ Medical packaging must strike a balance between offering robust physical and barrier properties to protect enclosed medical devices from microorganisms and external contaminants, while still facilitating the transmission of sterilizing agents such as ethylene oxide. Furthermore, oxygen plays a critical role in effective ventilation of the residual ethylene oxide, making oxygen transmission rate measurements an essential part of the study. Therefore, based on previous experiences with copolymers containing dilinoleic diol, we selected the composition of 70–30 wt% hard to soft segments for further exploration of their structure and properties in the context of future materials for medical sector. This specific ratio demonstrated a favorable combination of properties, including high flexibility with good mechanical performance, and dimensional stability. However, the potential for the sterile barrier system has not been explored yet, especially in the context of using aliphatic diols of variable chain length to change the distance between the spiral motifs of the carboxyls of FDCA and thus manipulate the final properties, and it will be discussed for the first time in this work. Therefore, the outcomes of this research hold significant promise for advancing the development of environmentally sustainable materials for medical sector. By expanding the range of polyester materials derived from renewable resources and providing comprehensive insights into the characteristics of furan-based block copolyesters, this study contributes to the field of materials science, fostering the creation of greener solutions.

## 2. Experimental

### 2.1 Materials

The following chemicals were purchased from Sigma-Aldrich (Poznan, Poland): 1,6-hexanediol (HDO; ≥99%), 1,12-dodecanediol (DDDO; ≥99%), diphenyl ether (DE; ≥99%). 1,8-Octanediol (ODO; ≥98%), and 1,10-decanediol (DDO; ≥99%) were acquired from Acros Organics (Geel, Belgium). Dimethyl 2,5-furandicarboxylate (DMFDCA; ≥99%) was obtained from Fluorochem (Lodz, Poland). Dilinoleic diol (DLD; ≥96.5%) (trade name: Pripol™ 2033) was obtained from Cargill Bioindustrials (Gouda, The Netherlands). Chloroform (≥98.5%) was purchased from Chempur (Piekary Slaskie, Poland) and methanol (≥99.8%) was purchased from Stanlab (Lublin, Poland). *Candida antarctica* lipase B (CAL-B) covalently immobilized on polyacrylate beads (300–500 µm; ≥95%, Fermase CALB™ 10 000), with a nominal activity of 10 000 PLU per g (propyl laurate units per gram dry weight) was acquired from Fermenta Biotech Ltd, Mumbai and Enzyme Catalyzed Polymers LLC (Akron, OH, USA). Before use, CAL-B was pre-dried under vacuum for 24 h at 40 °C, and diphenyl ether was stored over 4 Å molecular sieves. The rest of the chemicals were used as received.

### 2.2 CAL-B catalyzed polycondensation of DMFDCA with various diols

To obtain furan-based copolyesters, a two-step polycondensation reaction was conducted in diphenyl ether with varying temperatures. The procedure involved the following steps: initially, 2,5-DMFDCA, α,ω-aliphatic linear diol (α,ω-ALD), dilinoleic diol (DLD), and diphenyl ether (200 wt% of total monomers) were combined in a 100 ml three-necked round-bottom flask containing pre-dried CAL-B (10 wt% of total monomers). The mixture was magnetically stirred in an oil bath at 80 °C for 1 hour under an inert gas flow, aiming to achieve a homogeneous mixture. Subsequently, the temperature was increased to 95 °C, initiating the transesterification reaction. During this step, methanol was produced as a by-product and collected in a cold trap, which was adjusted to the neck of the round-bottom flask. This process lasted for 3 hours. After the transesterification, further oligomerization was carried out under a pressure of 600 torr for 21 hours. The application of a mild vacuum in this step created a controlled environment that facilitated efficient oligomerization and prevented monomer loss caused by evaporation. Finally, a polycondensation reaction was performed at 95 °C under reduced pressure of 2 torr for 24 hours, followed by an additional 48 hours at 140 °C.

After the polymerization, chloroform was added to the reaction flask to dissolve the final products and remove the catalyst *via* normal filtration. The obtained chloroform solution was then concentrated and added dropwise into excess of freezer-cold methanol (−20 °C) under continuous stirring. The obtained methanol solution with precipitated white polymer product was then filtered, washed three times with freezer-cold methanol, collected, and vacuum-dried at 40 °C for 24 h.

### 2.3 Nuclear magnetic resonance spectroscopy

^1^H and ^13^C NMR spectra were collected on a Bruker spectrometer (800 MHz, 10 s relaxation delay, 128 scans for ^1^H- and 700 MHz, 10 s relaxation delay, 2048 scans for ^13^C-NMR). Deuterated chloroform (CDCl_3_) was used to dissolve furan-based copolyesters.

### 2.4 The Fourier transform infrared (FTIR) spectroscopy

FTIR spectra were registered on a Bruker ALPHA spectrometer equipped with an attenuated total reflection (ATR) accessory. The spectra were recorded in a range of 400–4000 cm^−1^ and a resolution of 2 cm^−1^. Samples were vacuum-dried before measurement and 32 scans were performed for each specimen.

### 2.5 Size exclusion chromatography (SEC)

The number (*M*_n_) and weight (*M*_w_) averaged molecular weight, as well as dispersity index (*Đ*) of furan-based copolymers, were determined by size exclusion chromatography (SEC) using an Agilent 1200 modular HPLC series system with a refractive index detector. The system was equipped with two PLgel 5 µm MIXED-C columns (300 × 7.5 mm). Calibration was conducted on 12 polystyrene standards with masses (*M*_p_) in the range of 474–1 800 000 g mol^−1^. Measurements were performed at 35 °C. Chloroform (CHCl_3_) HPLC grade with a flow rate of 0.7 ml min^−1^ was used as the mobile phase. The specimens with a concentration of 3 mg ml^−1^ were filtered through a PTFE membrane with a 0.2 µm pore size before measurement. Data were recorded using the “ChemStation for LC” program and analyzed using the “ChemStation SEC Data Analysis Software”.

### 2.6 X-ray diffraction (XRD)

X-ray diffraction patterns of furan-based polymeric films were collected using an Empyrean PANalytical X-ray diffractometer using Cu Kα (*λ* = 0.154 nm) as the radiation source. In the 2*θ* range 10–60 with a step size of 0.026. The crystallinity index 
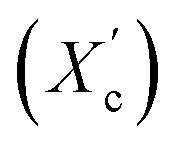
 was computed from the XRD profiles from the ratio between the crystalline peaks area (*A*_c_) and the area of total peaks (crystalline and amorphous) (*A*_t_). The degree of crystallinity 
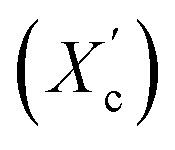
 was calculated from the XRD profiles by the ratio between the crystalline peaks area (*A*_c_) and diffraction area of all peaks (crystalline and amorphous) (*A*_t_) using the eqn (1).1
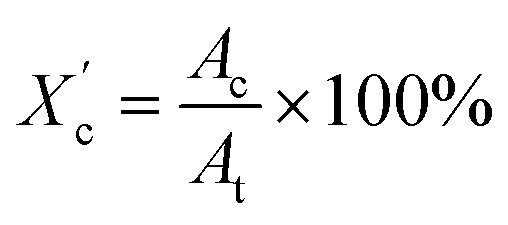


### 2.7 Digital holographic microscope

The microstructure of materials was evaluated after spontaneous crystallization using a Lyncée Tec DHM T1000 digital holographic transmission microscope. A Mach–Zehnder interferometer and 666 nm laser diode emitting linear polarized beam with a low illumination power of 200 µm cm^−2^ were used to achieve holographic off-axis geometry. Holograms were captured by a CCD camera (1024 × 1024 pixels, 30 fps) with magnification 10× (objective NA = 0.3; FOV = max 660 µm; no immersion). The lateral resolution was 0.1 µm and the axial resolution was below 1 nm. Data were analyzed using the Koala software of Lyncée Tec. Prior to measurement, samples were placed between two microscope slides, heated to about 20 °C above melting temperature, and cooled to room temperature. The samples were a few µm thick between the slides.

### 2.8 Thermal properties

Thermal studies were performed using differential scanning calorimetry (DSC). Measurements were performed on a polymer powder sealed in hermetic aluminum pans using a TA Instruments DSC Q2500 Discovery differential scanning calorimeter in the heating–cooling–heating cycles with the standard rate of 10 °C min^−1^. The measurements were conducted over the temperature range of −90 to 200 °C under a nitrogen atmosphere. The glass transition temperatures (*T*_g_) were taken as a midpoint of the transition during a second heating stage. The melting temperature (*T*_m_) and the crystallization temperature (*T*_c_) were verified as the maximum of the endothermic or exothermic peaks at the DSC thermographs, respectively. Furthermore, the enthalpy of melting (Δ*H*_m_) and crystallization (Δ*H*_c_) were established by the integration of the normalized area of the peaks related to a stated transition.^5^ The total crystalline phase content (*X*_c,tot_) and crystalline phase content in the hard segments (*X*_c,h_) were calculated using eqn (2) and (3), respectively.2
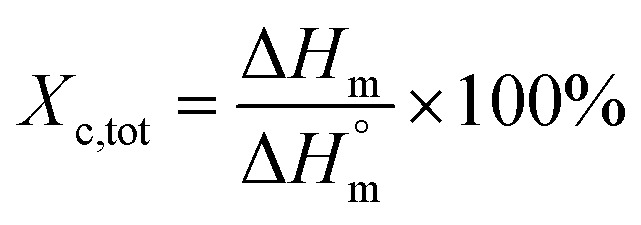
3
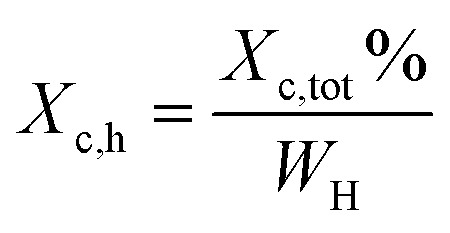
where *W*_H_ represents the weight content of the hard segments, Δ*H*_m_ is the melting enthalpy of the copolymer, and 
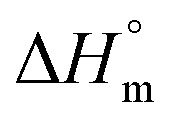
 is the melting enthalpy of fully crystalline PBA (135.0 J g^−1^) copolyester 
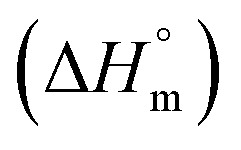
. For PHF 
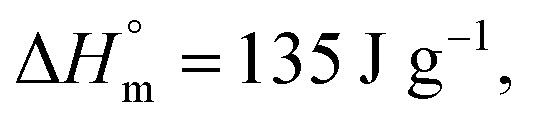
 for POF 
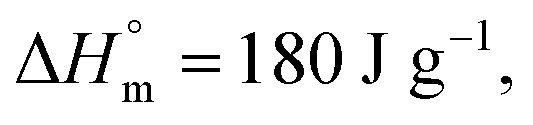
 for PDF 
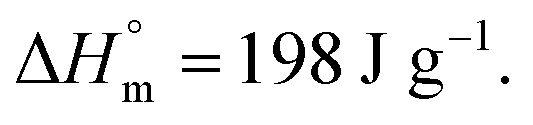
^6^ For PDDF 
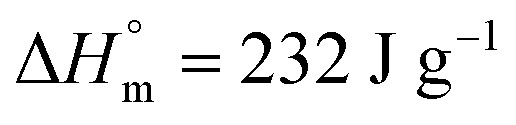
 – this value has been approximated by extrapolation of enthalpy of fusion of 100% crystalline PHF, POF, and PDF polyesters as a function of the number of carbon atoms within diol unit.^6^

### 2.9 Oxygen transmission rate (OTR)

The oxygen transmission rate was measured on 100 µm-thick polymer films using the OX-TRAN Model 2/10 (Mocon) in accordance with ASTM D3985. The area of the sample was 5 cm^2^. The tests were performed at a temperature of 23 °C and a relative humidity of 0%.

### 2.10 Dynamic mechanical analysis (DMTA)

To determine the storage modulus, samples for dynamic mechanical analysis (DMTA) were prepared by melt-pressing within a temperature range of 100 to 140 °C. The specimens had dimensions of 100 µm thickness, 1 mm width, and 50 mm length. Measurements were performed in tensile mode using the DMA Q800 apparatus from TA Instruments. The analysis was conducted at a constant frequency of 1 Hz, a heating rate of 2 °C min^−1^, and an amplitude of 60.

## 3. Results and discussion

**Scheme 1:**
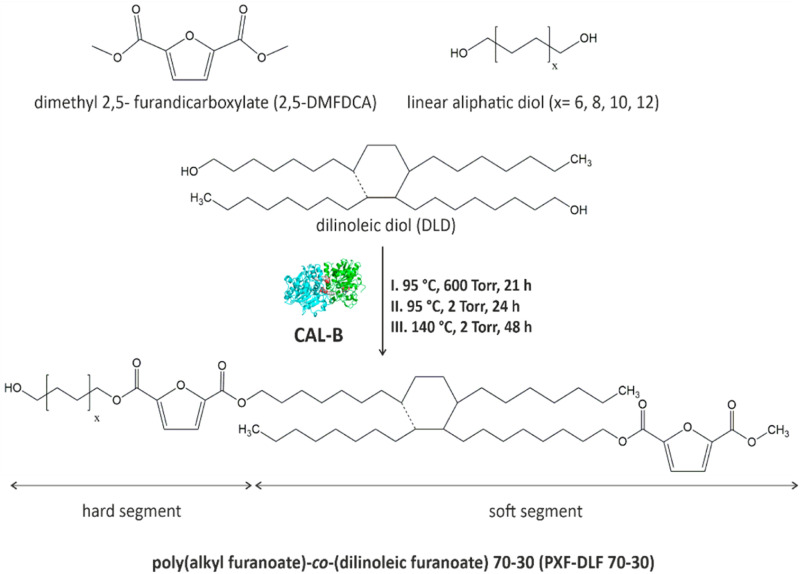
General scheme of CAL-B catalyzed synthesis of poly(alkyl furanoate)-*co*-(dilinoleic furanoate) copolyesters *via* the temperature-varied two-stage method in diphenyl ether.

New entirely bio-based series of copolymers containing poly(alkyl furanoate) as a hard segment and poly(dilinoleic furanoate) (DLF) as a soft segment with a 70–30 wt% ratio, respectively, were synthesized *via* direct transesterification of 2,5-DMFDCA with different diols (*C* = 6, 8, 10 and 12) and consecutive polycondensation following temperature varied two-stage method in diphenyl ether, as illustrated in Scheme 1.

As can be concluded from the literature, enzyme catalysts reach the highest catalytic efficiency in temperatures below 100 °C, however, due to the high melting temperatures of furan-based copolyesters and poor solubility of the final products, it was decided to perform the reaction at higher temperature (140 °C). The immobilized form of CAL-B can still maintain its catalytic reactivity in elevated temperatures even up to 150 °C,^6,37^ which is above the denaturation point of CAL-B protein which was established experimentally to be 62 °C.^38^ On the one hand, this is an advantage because the syntheses can be carried out at higher temperatures, which allows for catalyzing a wide range of reactions, but on the other hand, it should be borne in mind that most probably the depletion of enzyme activity, in this case, is faster which directly translates into the limited reusability. Nevertheless, performed synthesis resulted in four different furan-based copolymers with high reaction yields (>89%). The aliphatic diols used to create hard segments were α,ω-aliphatic linear diols (α,ω-ALD) possessing two primary hydroxyl groups: 1,6-HDO, 1,8-ODO, 1,10-DDO, 1,12-DDDO where the number of carbon atoms located between the two hydroxyl groups is 6, 8, 10, and 12, respectively. Dilinoleic diol (1,36-DLD) possessing 36 carbon atoms was used to create the soft segment due to its long aliphatic chain and low cohesion energy (2.85 kJ mol^−1^) which gives the ability of free rotation of carbon–carbon bonds. Depending on the α,ω-ALD used: 1,6-HDO, 1,8-ODO, 1,10-DDO, 1,12-DDDO, copolymers were denoted as PHF-DLF, POF-DLF, PDF-DLF, PDDF-DLF, respectively. Therefore, as the molecular mass and chemical structure of the DLF soft segment were constant, the changes in the composition of the copolymers were consistent with the changes in the hard segments length.

Enzymatic synthesis was also performed using 1,4-butanediol (1,4-BDO), however, due to the poor reaction yield (11%) and low values of number and weight average molecular weights (1100 and 1300 g mol^−1^, respectively), this material was not subjected to further analysis.

**Scheme 2:**
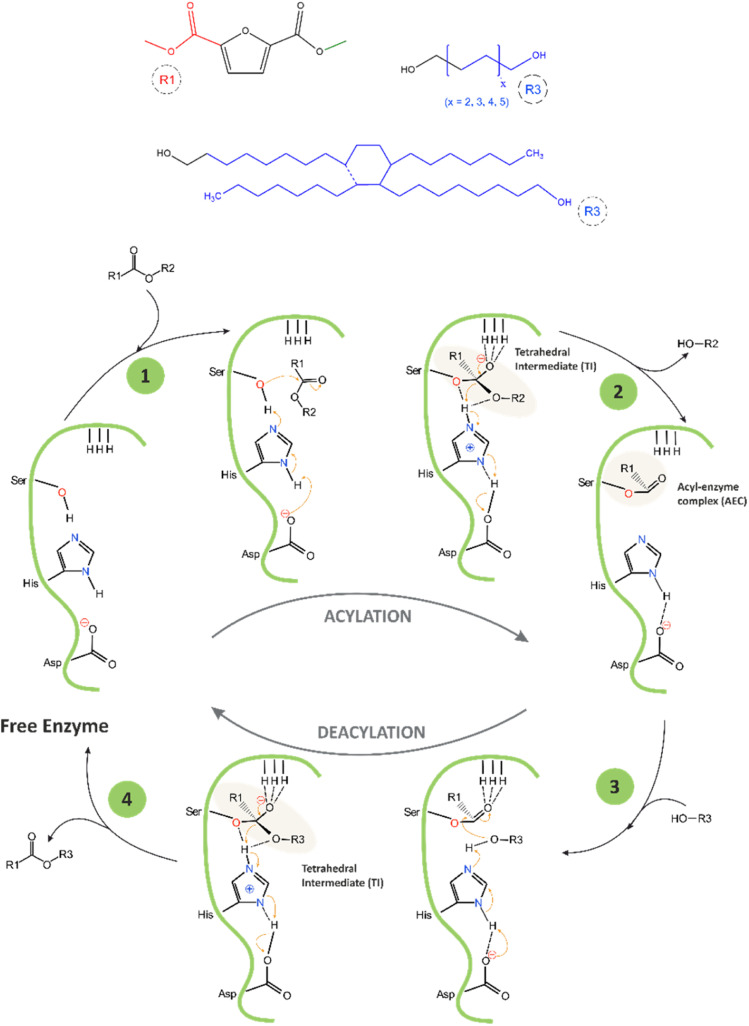
Proposed mechanism of CAL-B-catalyzed transesterification of DMFDCA with α,ω-ALD, and DLD.

The proposed mechanism of the catalytic cycle of CAL-B-catalysed transesterification of DMFDCA with α,ω-ALD, and DLD based on the mechanism described by Puskas and García,^22,39^ is presented in Scheme 2. The catalytic triad of CAL-B consists of three amino acids, serine (Ser105), histidine (His224), and aspartic acid (Asp187), arranged in a specific spatial orientation within the enzyme's active site. Another important structural feature of the active site of CAL-B is the oxyanion hole, which helps facilitate its catalytic activity. The oxyanion hole is a pocket-like structure located above the catalytic triad that stabilizes the negative charge that develops on the carbonyl oxygen of the substrate during the reaction by using three hydrogen bonds. One bond is provided by glutamine (Gln106), and two are provided by threonine (Thr40).

During the first step of the reaction, the primary alcohol from Ser105, which is the nucleophilic centre of the catalytic triad, attacks the ester bond of DMFDCA, forming the first tetrahedral intermediate (TI) stabilized by the three hydrogen bonds located in the oxyanion hole. During this process, the imidazole group of His224 acts as a general base and accepts a proton from Ser105, thereby activating it and enhancing its nucleophilicity, while Asp187 stabilizes the His224 residue by forming a hydrogen bond with it. In the second step, methanol is released, and the acyl–enzyme complex (AEC) is formed. Methanol is continuously removed *via* evaporation, making the reaction irreversible. In the third step, the hydroxyl group from α,ω-ALD, and DLD interacts with AEC, creating the second tetrahedral intermediate, again stabilized by the oxyanion hole. In the final step, the enzyme is deacylated, and the ester product is formed.

**Fig. 2:**
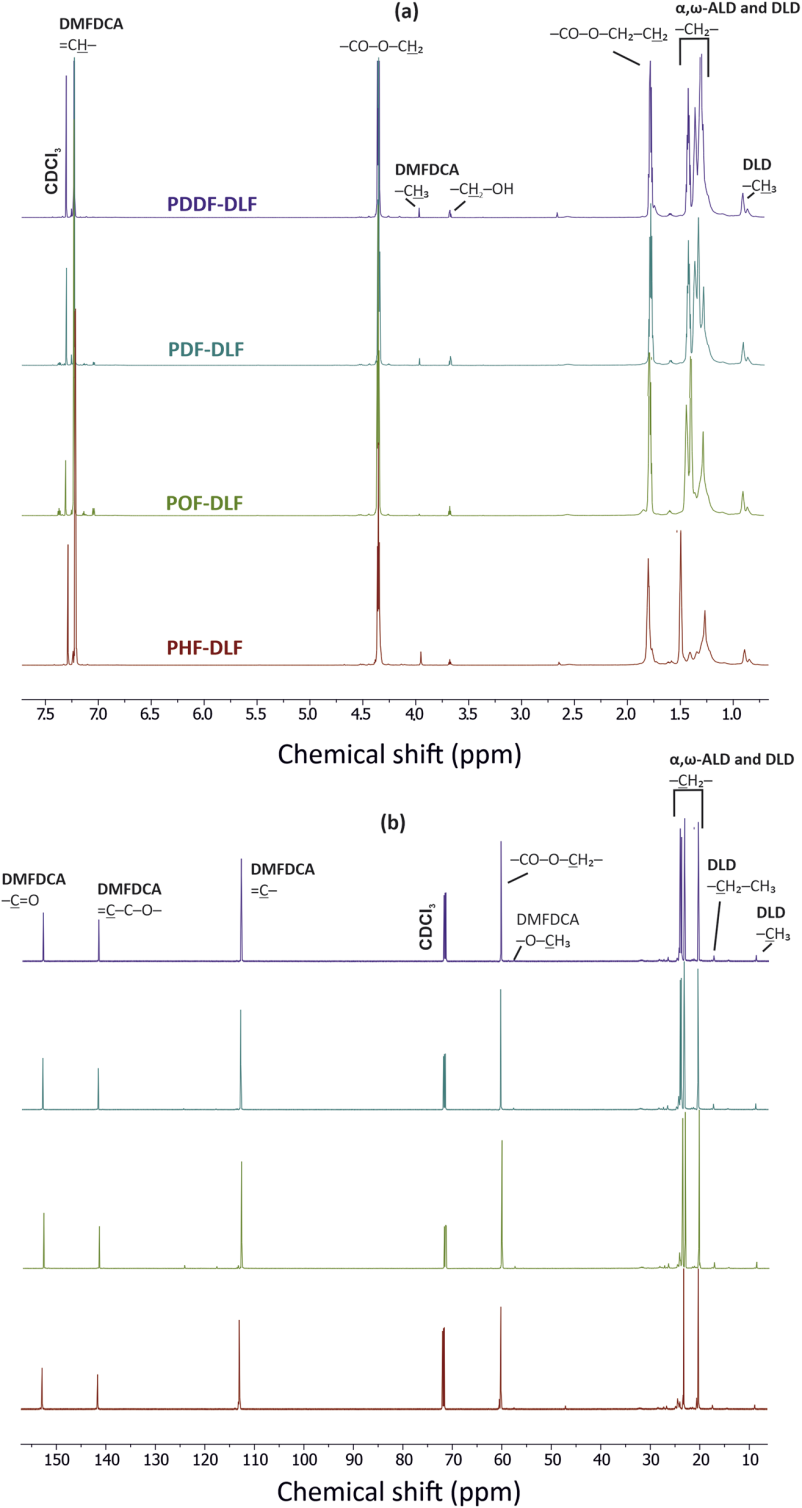
(a) ^1^H NMR and (b) ^13^C NMR spectra of the furan-based copolyesters.

**Fig. 3:**
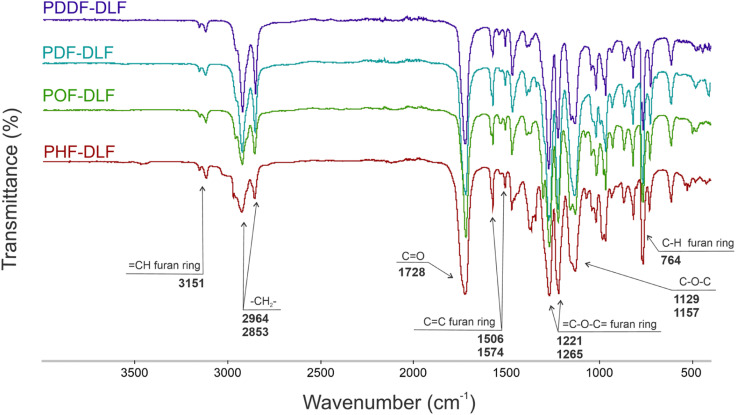
FTIR spectra of furan-based copolyesters.

In order to validate the proposed mechanism and assess the chemical structure of furan-based copolyesters, we conducted NMR and FTIR analyses (Fig. 2 and 3). Detailed ^1^H, ^13^C NMR, and FTIR spectra of representative furan-based copolyesters are provided in the ESI (Fig. S1–S4 for NMR and Fig. S5–S8 for FTIR).† Additionally, the calculation of chemical composition and molecular weight has been included in the ESI and summarized in Table S1.† For further clarity, detailed NMR assignments are provided as follows:

### 3.1 Poly(hexamethylene furanoate)-*co*-(dilinoleic furanoate) (PHF-DLF)

^1^H NMR (800 MHz, CDCl_3_, ppm): 7.22 (2H, –CH̲=, furan ring), 4.35 (4H, –CO–O–CH̲_2_, from 1,6-HDO and DLD), 3.95 (6H, –O–CH̲_3_, end group from DMFDCA), 3.68 (4H, –CH̲_2_–OH, end group from 1,6-HDO and DLD), 1.80 (4H, –CO–O–CH_2_–CH̲_2_–, from 1,6-HDO and DLD), 1.49 (–CH̲_2_– internal methylene groups from 1,6-HDO), 1.41–1.27 (–CH_2_– internal methylene groups from DLD), 0.88 (6H, –CH_2_–CH̲_3_, end groups from DLD); ^13^C NMR (700 MHz, CDCl_3_, ppm): 152.88 (–C̲=O, furan), 141.67 (=C̲–C–O–, furan ring), 113.06 (–C=, furan ring), 60.18 (–CO–O–C̲H_2_–), 57.55 (–CH_2_–OH, end group from 1,6-HDO and DLD), 47.13 (–O–CH_3_–, end group from DMFDCA), 24.48–17.46 (–CH_2_–, internal methylene groups from 1,6-HDO and DLD), 8.89 (–CH_2_–C̲H_3_, end groups from DLD).

### 3.2 Poly(octamethylene furanoate)-*co*-(dilinoleic furanoate) (POF-DLF)

^1^H NMR (800 MHz, CDCl_3_, ppm): 7.19 (2H, –CH̲=, furan ring), 4.31 (4H, –CO–O–CH̲_2_, from 1,8-ODO and DLD), 3.92 (6H, –O–CH̲_3_, end group from DMFDCA), 3.64 (4H, –CH̲_2_–OH, end group from 1,8-ODO and DLD), 1.75 (4H, –CO–O–CH_2_–CH̲_2_–, from 1,8-ODO and DLD), 1.40 (–CH_2_– internal methylene groups from 1,8-ODO), 1.35–1.24 (–CH̲_2_– internal methylene groups from 1,8-ODO and DLD), 0.86 (6H, –CH_2_–CH̲_3_, end groups from DLD); ^13^C NMR (700 MHz, CDCl_3_, ppm): 152.92 (–C̲=O, furan), 141.71 (=C̲–C–O–, furan ring), 112.96 (–C=, furan ring), 60.44 (–CO–O–C̲H_2_–), 57.81 (–CH_2_–OH, end group from 1,8-ODO and DLD), 24.48–17.46 (–CH_2_–, internal methylene groups from 1,8-ODO and DLD), 8.89 (–CH_2_–C̲H_3_, end groups from DLD).

### 3.3 Poly(decamethylene furanoate)-*co*-(dilinoleic furanoate) (PDF-DLF)

^1^H NMR (800 MHz, CDCl_3_, ppm): 7.19 (2H, –CH̲=, furan ring), 4.31 (4H, –CO–O–CH̲_2_, from 1,10-DDO and DLD), 3.93 (6H, –O–CH̲_3_, end group from DMFDCA), 3.64 (4H, –CH̲_2_–OH, end group from 1,10-DDO and DLD), 1.74 (4H, –CO–O–CH_2_–CH̲_2_–, from 1,10-DDO and DLD), 1.39 (–CH_2_– internal methylene groups from 1,10-DDO), 1.32–1.27 (–CH̲_2_– internal methylene groups from 1,10-DDO and DLD), 0.87 (6H, –CH_2_–CH̲_3_, end groups from DLD); ^13^C NMR (700 MHz, CDCl_3_, ppm): 152.94 (–C̲=O, furan), 141.73 (=C̲–C–O–, furan ring), 112.96 (–C=, furan ring), 60.44 (–CO–O–C̲H_2_–), 57.81 (–CH_2_–OH, end group from 1,10-DDO and DLD), 47.16 (–O–CH_3_–, end group from DMFDCA), 28.49–17.46 (–CH_2_–, internal methylene groups from 1,10-DDO and DLD), 8.89 (–CH_2_–C̲H_3_, end groups from DLD).

### 3.4 Poly(dodecamethylene furanoate)-*co*-(dilinoleic furanoate) (PDDF-DLF)

^1^H NMR (800 MHz, CDCl_3_, ppm): 7.19 (2H, –CH̲=, furan ring), 4.32 (4H, –CO–O–CH̲_2_, from 1,12-DDDO and DLD), 3.93 (6H, –O–CH̲_3_, end group from DMFDCA), 3.64 (4H, –CH̲_2_–OH, end group from 1,12-DDDO and DLD), 1.74 (4H, –CO–O–CH_2_–CH̲_2_–, from 1,12-DDDO and DLD), 1.40 (–CH_2_– internal methylene groups from 1,12-DDDO), 1.35–1.25 (–CH̲_2_– internal methylene groups from 1,12-DDDO and DLD), 0.87 (6H, –CH_2_–CH̲_3_, end groups from DLD); ^13^C NMR (700 MHz, CDCl_3_, ppm): 152.95 (–C̲=O, furan), 141.74 (=C̲–C–O–, furan ring), 112.94 (–C=, furan ring), 60.47 (–CO–O–C̲H_2_–), 57.85 (–CH_2_–OH, end group from 1,12-DDDO and DLD), 47.12 (–O–CH_3_, end group from DMFDCA), 28.50–17.46 (–CH_2_–, internal methylene groups from 1,12-DDDO and DLD), 8.89 (–CH_2_–C̲H_3_, end groups from DLD).

The collected ^1^H and ^13^C NMR spectra verified the chemical structure of copolyesters. Minor signals ascribed to the end-groups of α,ω-ALD appeared at the 3.68–3.64 ppm region clearly indicating that enzymatic synthesis was successfully performed and products with relatively high molecular weight were formed. On the basis of the proton signal integration ratio of signals characteristic for hard and soft segments, it was possible to calculate the real segmental composition (see ESI†). Calculated values are close to those established theoretically which indicates good control over the process. However, as can be noticed from Table 1, the content of hard segments increases with an increasing number of carbon atoms within α,ω-ALD. Most probably this phenomenon is related to the fact that under applied vacuum and high reaction temperatures, some quantities of diols possessing lower boiling points were removed from the reaction mixture. Since the boiling point values increase with the increasing length of the diol chain, PDDF-DLF material possesses greater hard segment content in comparison to PHF-DLF copolyester (70.7 *vs.* 67.5 wt%, respectively).

**Table 1:** The composition of furan-based copolyesters was determined from ^1^H NMR and SEC^a^

Copolymer	Composition		^1^H NMR^b^	SEC^c^		
	Theoretical wt% [mol%]	Calculated^b^ wt% [mol%]	*M*_n_ [g mol^−1^]	*M*_n_ [g mol^−1^]	*M*_w_ [g mol^−1^]	*Đ*
PHF-DLF	70/30 [86.6/13.4]	67.5/32.5 [85.2/14.8]	17 600	5700	10 700	1.9
POF-DLF	70/30 [85.3/14.7]	68.1/31.9 [84.2/15.8]	19 500	13 700	40 500	3.0
PDF-DLF	70/30 [84.0/16.0]	70.0/30.0 [84.0/16.0]	23 900	19 000	42 600	2.2
PDDF-DLF	70/30 [82.8/17.2]	70.7/29.3 [83.2/16.8]	34 800	20 600	45 700	2.2

a *M*_n_ – number average molecular mass, *M*_w_ – weight average molecular mass, *Đ* – dispersity index.

b Values calculated from ^1^H NMR.

c Values determined from SEC.

The chemical structure of furan-based copolymers was also validated by ATR-FTIR analysis (Fig. 3). The characteristic absorption bands of the obtained furan-based copolyesters are ascribed as follows: ATR-FTIR (cm^−1^): 3151 and 3118 (=C–H stretching vibrations in the furan ring), 2922 and 2855 (C–H stretching), 1718 (C=O stretching), 1573 and 1506 (C=C stretching vibrations in the furan ring) 1471 and 1390 (–CH– deformations and wagging peaks), 1269 and 1130 (C–O–C asymmetric and symmetric stretching), 1221 and 1016 (=C–O–C= stretching vibrations in the furan ring), 966, 867, and 764 (C–H bending vibrations in the furan ring).

In order to determine the number-averaged (*M*_n_) and weight-averaged (*M*_w_) molecular weights of the copolyesters, size exclusion chromatography (SEC) analysis was conducted. SEC curves presenting the molar mass distribution of the copolyesters can be found in the ESI, specifically in Fig. S9.† Obtained results revealed that the two-stage enzymatic polymerization of DMFDCA with α,ω-ALD possessing longer chain length yields furan-based copolyesters with higher molecular weight. This phenomenon is in line with the research conducted by Jiang *et al.* and Nasr *et al.* in which they performed a synthesis of furanic–aliphatic polyesters using aliphatic diols differing in chain length and showed that greater *M*_n_ and *M*_w_ values were achieved for longer-chain α,ω-ALD.^6,34^ As presented in Table 1, molecular weights of copolymers gradually increase with an increasing number of carbon atoms within the α,ω-ALD structure. The *M*_n_ and *M*_w_ values from 5700 and 10 700 g mol^−1^ increased to 13 700 and 40 500 g mol^−1^, respectively, while changing the number of carbon atoms from 6 to 8. Moreover, further increasing the chain length to 12 enabled to obtain *M*_n_ and *M*_w_ values of 20 600 and 45 700 g mol^−1^, respectively.

There are two potential explanations for this phenomenon. Firstly, diols with longer alkylene chains have increased enzymatic reactivity, leading to the production of polyesters with higher degrees of polymerization.^40^ Secondly, it was proven that the solubility of the furanic–aliphatic polyesters decreases while using α,ω-ALD with shorter chains.^6^ Additionally, different melting temperatures (*T*_m_) of copolyesters were noticed. PHF-DLF/POF-DLF materials are characterized by higher *T*_m_ (130 and 127 °C, respectively) than that of PDF-DLF/PDDF-DLF materials (96 and 92 °C, respectively) (Table 4), as well as by the synthesis temperature applied during the I and II stage (95 °C). Therefore, during the reaction of DMFDCA with 1,6-HDO/1,8-ODO, low molecular weight products could be precipitated from the reaction media due to their high *T*_m_, until the temperature was raised to 140 °C in the final III temperature stage. As a result, polymerization efficiency was lower since CAL-B was unable to reach the polymers most of the reaction time, hence, lower molecular weight values were obtained. On the other hand, enzymatic polymerization of DMFDCA with 1,10-DDO/1,12-DDDO diols which exhibits lower *T*_m_ (95 and 91 °C, respectively) proceeded constantly with the same efficiency since no product precipitated out from the reaction media, and the access of CAL-B to polymers was not hindered. As a result, high-molecular-weight copolymers were obtained. Furthermore, when examining the dispersity index values of furan-based copolyesters, they range from 1.9 to 3.0, which is characteristic of polyesters produced through step-growth polycondensation reactions. However, the enzyme utilized as a biocatalyst in this study might also contribute to the variations in dispersity values since they display selectivity towards specific monomers or reaction pathways, which can influence the overall polymerization process.

Additionally, based on ^1^H NMR spectra and integrals of signals corresponding to CH̲_3_ protons arising from DLD (6H), CH̲_2_ protons arising from α,ω-ALD diols (4H), and CH̲_2_–OH protons arising from α,ω-ALD end-groups, number averaged molecular weight (*M*_n_) of copolyesters were calculated following the method described in our previous work^26^ and presented in Table 1.^25^ Herein, the calculated values differ from those obtained on the SEC basis, however, we are still observing the same trend that *M*_n_ values increase with an increasing number of carbon atoms within α,ω-ALD.

**Fig. 4:**
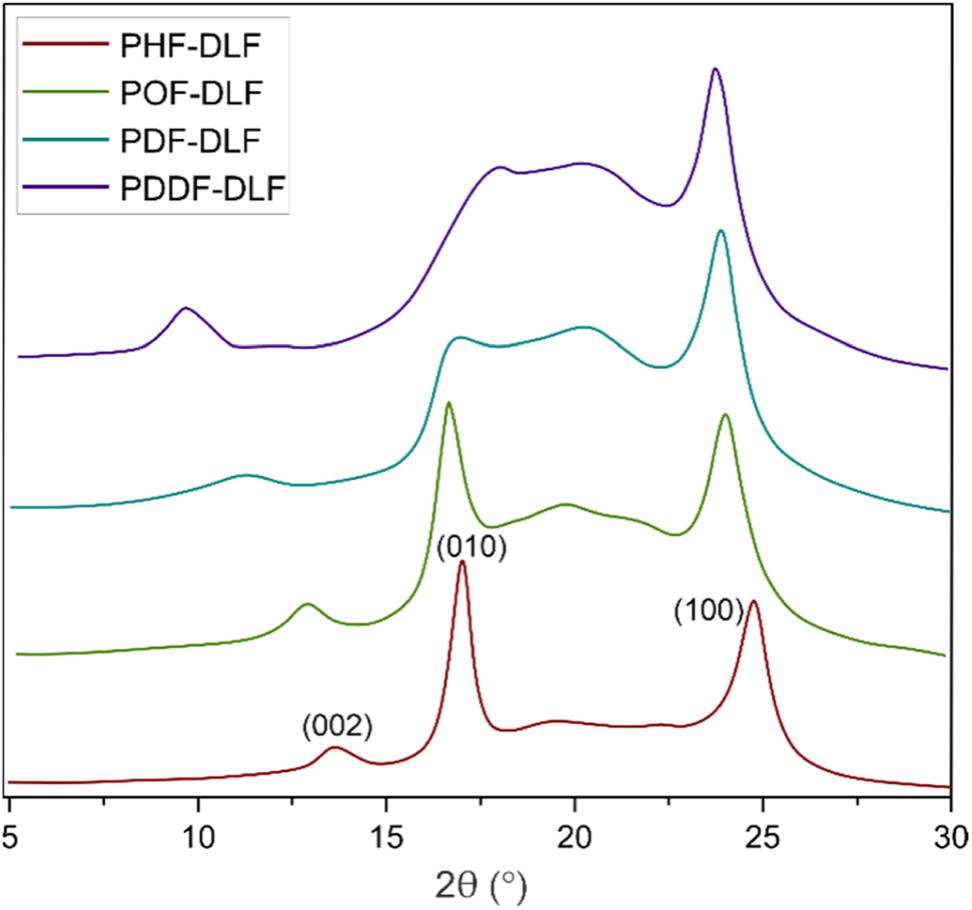
XRD spectra of furan-based copolyesters.

In order to evaluate the effects of diol chain length on the crystalline structure of furan-based copolyesters, X-ray diffraction (XRD) analysis was conducted on 100 µm-thick melt-pressed polymer films. Performed studies revealed that furan-based copolyesters are semicrystalline materials. As shown in Fig. 4 and Table 2 the diffraction pattern of the tested PHF-DLF was characterized by a low intense peak at 2*θ* of 13.73° (*d* = 6.44 Å) and two strong intense peaks at 2*θ* of 17.10 (*d* = 5.18 Å) and 24.86° (*d* = 3.58 Å). Three similar diffraction peaks can be observed in case of POF-DLF at 12.99 (*d* = 6.81 Å), 16.78 (*d* = 5.28 Å), 24.12° (*d* = 3.69 Å), and also in case of PDF-DLF copolymer which XRD patterns appeared at 11.31 (*d* = 7.81 Å), 17.20 (*d* = 5.15 Å), and 23.91° (*d* = 3.72 Å). Moreover, for PDDF-DLF copolymer XRD patterns, appeared at 9.74 (*d* = 9.08 Å), 18.14 (*d* = 4.88 Å), and 23.81° (*d* = 3.73 Å). According to the presented data and the information provided in the literature,^6,41^ we assume that the analyzed furan-based copolymers crystallize into β-form crystals, where the three diffraction peaks can be marked as the lattice plan of β(002), β(010), and β(100), respectively. In the case of PDF-DLF and PDDF-DLF intensity of peaks that appeared at region 17.20 (in PDF-DLF) and 18.14° (in PDDF-DLF) are getting less pronounced in comparison to PHF-DLF and POF-DLF materials where similar peak appeared in 2*θ* of 17.10 and 16.78, respectively. This phenomenon suggests that the macromolecular order in PHF-DLF and POF-DLF copolyesters containing shorter diols is higher. Moreover, the rest of the main diffraction peaks mentioned above shifted to lower values when the diol chain length within hard segments increased. As a result, corresponding spacing between the planes in the atomic lattice (*d*) increased as well, which is quite expected as more space is needed to create the same crystal lattice if the alkylene chain of the diol is longer.

**Table 2:** XRD analysis of the obtained furan-based copolymers^a^

Copolymer	Crystal type	2*θ* (°)	*d* (Å)	Lattice plane	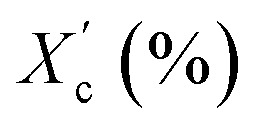
PHF-DLF	Triclinic β-form	13.73	6.44	β(002)	56.5
		17.10	5.18	β(010)	
		24.86	3.58	β(100)	
POF-DLF	Triclinic β-form	12.99	6.81	β(002)	50.0
		16.78	5.28	β(010)	
		24.12	3.69	β(100)	
PDF-DLF	Triclinic β-form	11.31	7.81	β(002)	42.3
		17.20	5.15	β(010)	
		23.91	3.72	β(100)	
PDDF-DLF	Triclinic β-form	9.74	9.08	β(002)	41.1
		18.14	4.88	β(010)	
		23.81	3.73	β(100)	

a *d* – spacing between the planes in the atomic lattice.

**Fig. 5:**
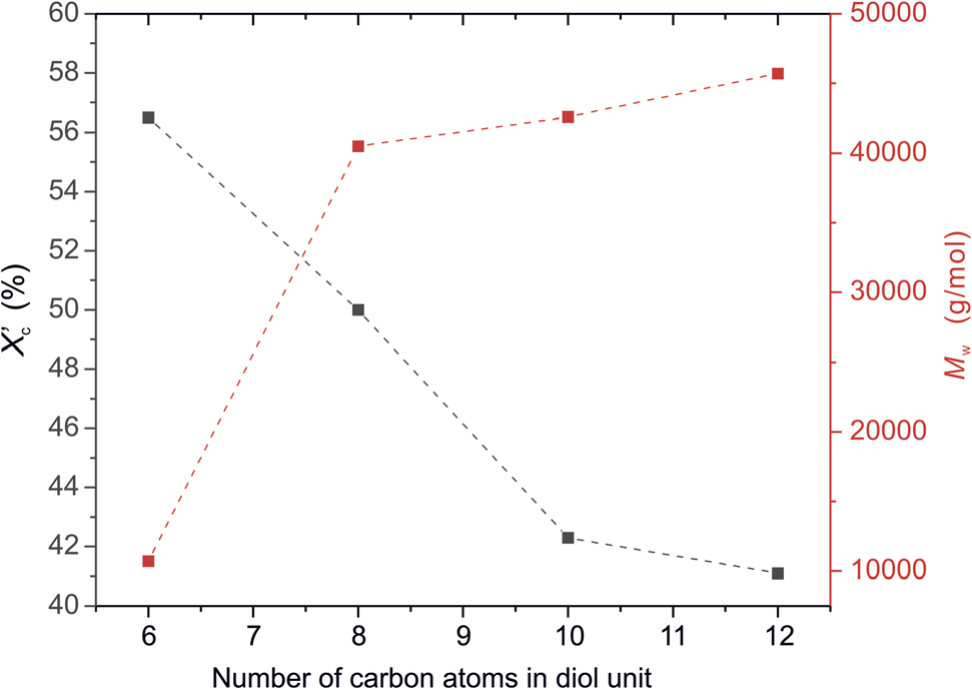
The degree of crystallinity 
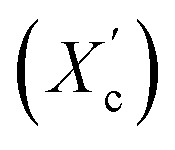
 and weight average molecular weight (*M*_w_) of furanic–aliphatic polyesters as a function of the number of carbon atoms in α,ω-aliphatic linear diol (α,ω-ALD) unit.

Moreover, the degree of crystallinity 
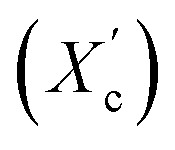
 was calculated from the XRD profiles. As shown in Fig. 4, the relative area of the amorphous halo increased while changing the number of carbon atoms within α,ω-ALD from 6 to 8, 10, or 12, and as a result, the degree of crystallinity 
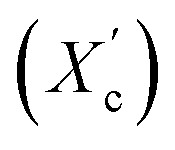
 decreased. The explanation for this phenomenon is the fact that chain regularity decreases and the ability to form crystal structures is hindered. Additionally, as evident from Fig. 5, it also may be correlated with weight averaged molecular mass (*M*_w_). The 
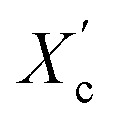
 values are decreasing with increasing *M*_w_ values because the mobility of polymer chains decreases faster with greater *M*_w_, and the entanglement of polymer chains increases quickly in parallel.

**Fig. 6:**
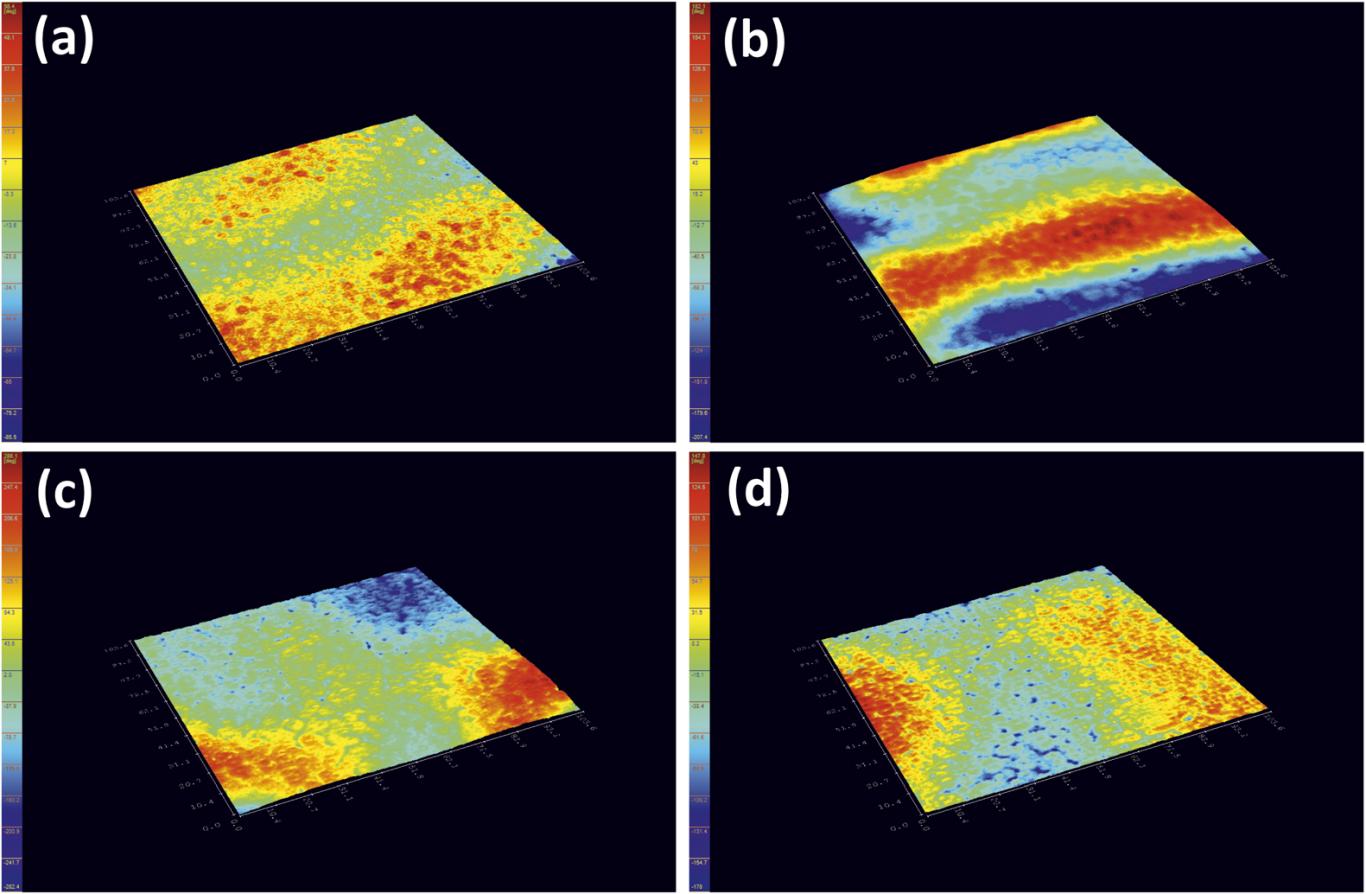
Holographic images of a crystallized layer of (a) PHF-DLF, (b) POF-DLF, (c) PDF-DLF, and (d) PDDF-DLF copolyester.

Furthermore, a digital holographic microscope was used to observe and record the crystallization behavior of furan-based copolyesters. Phase images were analyzed, which allow for the measurement of changes in the optical path (refractive index and geometrical path) and density distribution within the sample (see Fig. S10 in ESI†). By analyzing the profiles along the green lines visible in the phase images, we were able to measure differences in the phase change (Δ*φ*) when the laser beam passed through crystallites *versus* when it passed through amorphous areas. The images show spherulite formation when the sample of furan-based copolyester was cooled down to room temperature. The red color in Fig. 6 corresponds to the greatest phase change of the beam passing through the sample, and the dark blue color corresponds to the least change. The sample thickness was about a few microns.

The recorded holographic images and profiles along the green lines reveal that the spherulite diameters for furan-based copolymers fall within the range of 4–8 µm (Table 3). In most cases, the dimensions of the spherulites within the sample are the same, hence the assumption that the crystallization process probably occurs simultaneously in the entire volume of the sample. Additionally, the phase change between the crystalline and amorphous phase was analyzed and it was observed that the Δ*φ* values were at least 50% smaller for the PHF-DLF and POF-DLF copolymers (38 and 40 degrees, respectively) in comparison to the PDF-DLF and PDDF-DLF copolymers. This may indicate that these copolymers have a higher degree of crystallinity or more homogeneous distribution of crystallites in the amorphous phase, which is in accordance with results obtained from DSC and XRD measurements where a higher degree of crystallinity (*X*_c_ and 
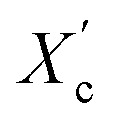
) were recorded for PHF-DLF and POF-DLF copolymers.

**Table 3:** Spherulites diameter and roughness of the sample (*S*_a_) after cooling from melt

Copolymer	Spherulite diameter [µm]	Sample roughness (*S*_a_)
PHF-DLF	4.5	24
POF-DLF	4.0	37
PDF-DLF	6.0	63
PDDF-DLF	8.0	42

Additionally, it was observed that the density heterogeneity of furan-based copolyesters underwent changes during spontaneous cooling with a decreasing exponentially temperature. The surface roughness parameter (*S*_a_) was calculated to evaluate the degree of homogeneity in the crystallization process. The results, as summarized in Table 3, showed that the crystallization process was homogenous throughout the whole volume in the case of the PHF-DLF copolymer, as evidenced by the lowest *S*_a_ value. However, as the number of atoms in the diol structure increased, the *S*_a_ value also increased, suggesting a less homogenous crystallization process. In particular, the greatest value of *S*_a_ was recorded for the PDF-DLF copolymer, indicating that spherulites crystallized at different depths, leading to an increase in the difference of the optical way. These findings indicate that the diol structure has a significant impact on the homogeneity of the crystallization process and have important implications for the design and development of new materials with specific properties. It is also important from a processing point of view since a homogenous crystallization process in polymeric materials can result in a uniform product with consistent properties, while a less homogenous crystallization process can lead to variations in the properties of the final product. Fast crystallization rates can reduce the processing time and allow for the production of materials with improved mechanical and thermal properties. On the other hand, slow crystallization rates can allow for better control over the structure of the crystalline material and may result in materials with improved optical properties. In general, the crystallization rate and the homogeneity of the crystallization process play important roles in the processing of polymeric materials and can influence the final properties of the product.

**Fig. 7:**
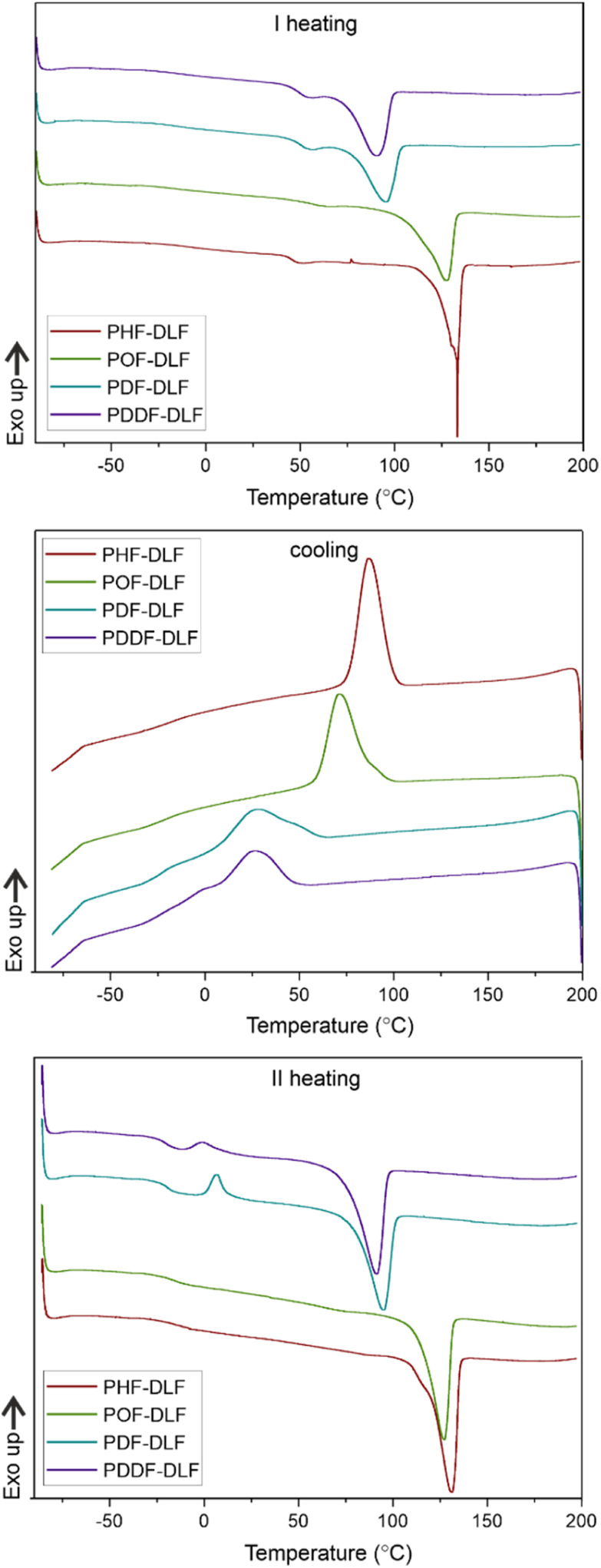
DSC first heating, cooling, and second heating thermograms of furan-based copolymer series.

**Fig. 8:**
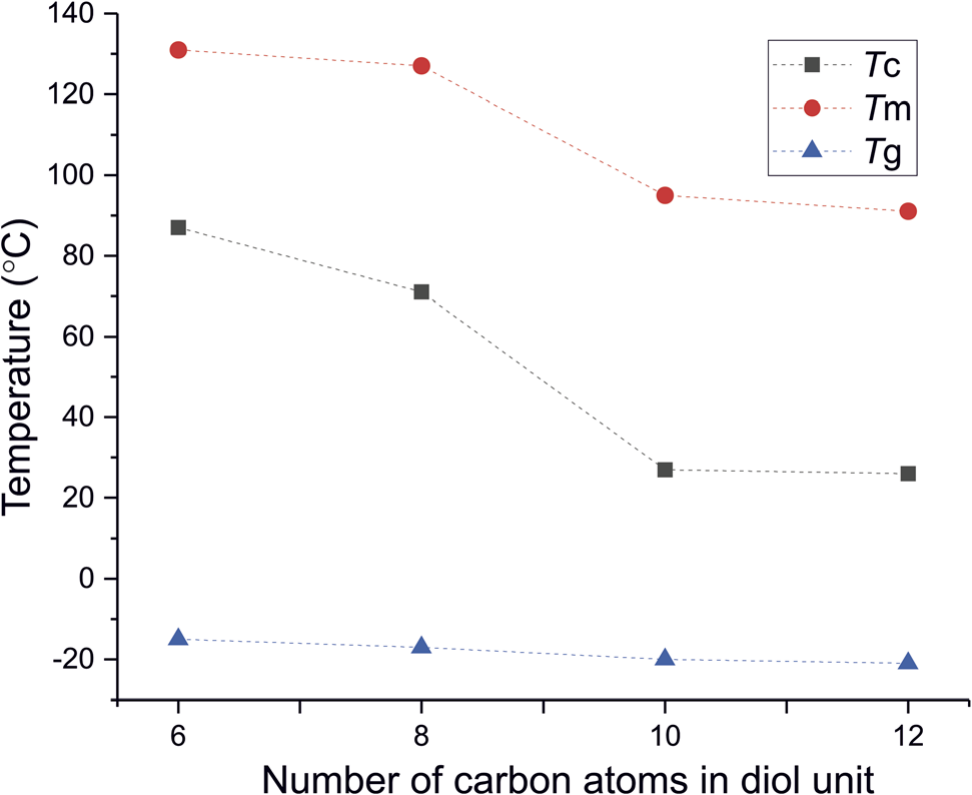
The crystallization (*T*_c_), melting (*T*_m_), and glass transition temperature (*T*_g_) of furan-based copolyesters as a function of the number of carbon atoms in α,ω-aliphatic linear diol (α,ω-ALD) unit.

**Fig. 9:**
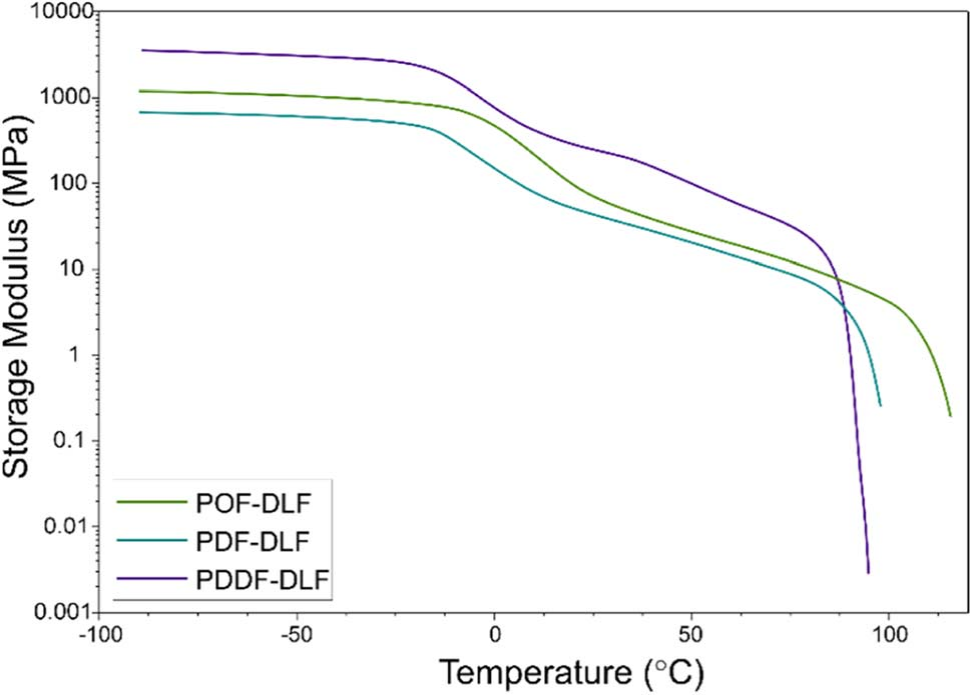
The dependence of storage modulus on temperature for furan-based copolymers.

DSC analysis has been performed to investigate the relationship between crystalline and thermal effects for furan-based copolyesters consisting of α,ω-ALD differing in chain length. Detailed results of the DSC analysis can be found in the ESI (Fig. S11–S14).† As can be seen from Fig. 7, 8 and Table 4 the thermal transition temperatures are governed by the chain length of α,ω-ALD which were used as a building block to create hard segments within furan-based copolyesters. Performed studies revealed that copolyesters exhibit specific phase-separated microstructure consisting of a crystalline phase, created by the ester hard segments and an amorphous phase formed by a mixture of the soft segments containing long aliphatic dilinoleic diol unit and hard segments which were not involved in crystallites formation. As evident from Fig. 8, 9 and Table 4 glass transition (*T*_g_), melting (*T*_m_), and crystallization (*T*_c_) temperature as well as crystallization degree (*X*_c_) were observed to decrease steadily with an increasing number of carbon atoms within the α,ω-ALD.

**Table 4:** DSC results for furan-based copolymer series^a^

Copolymer	PHF-DLF	POF-DLF	PDF-DLF	PDDF-DLF
*T*_g_ [°C]	−15	−17	−20	−21
Δ*C*_p_ [J g^−1^ °C^−1^]	0.243	0.288	0.281	0.343
*T*_c_ [°C]	87	71	27	26
Δ*H*_c_ [J g^−1^]	48.29	41.85	30.77	34.86
*T*_cc_ [°C]	—	—	7	−1
Δ*H*_cc_ [J g^−1^]	—	—	4.77	2.76
*T*_m_ [°C]	131	127	95	91
Δ*H*_m_ [J g^−1^]	44.29	38.24	34.40	36.43
*X*_c_ [%]	32.8	21.2	17.4	15.7
*X*_c,h_ [%]	48.6	31.1	24.6	22.2
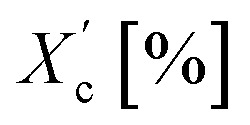	56.5	50.0	42.3	41.1

a *T*_g_ – glass transition temperature; Δ*C*_p_ – heat capacity at *T*_g_; Δ*H*_m_ – melting enthalpy; *T*_m_ – melting temperature; *T*_c_ – crystallization temperature; Δ*H*_c_ – crystallization enthalpy; *T*_cc_ – cold crystallization temperature; Δ*H*_cc_ – cold crystallization enthalpy; *X*_c_ – total crystalline phase content in the polymer calculated from DSC. *X*_c,h_ – crystalline phase content in hard segments calculated from DSC. 
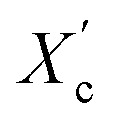
 – total crystalline phase content in the polymer calculated from XRD.

This effect can be easily explained since monomers with longer aliphatic chains were introduced to copolyester structure which on the other hand improves material flexibility, as reflected by a decrease of *T*_g_ values (from −15 to −21 °C) but on the other, hinders the ability to form crystallites, as reflected by the decrease of *X*_c_ and Δ*H*_m_. It is also evident from Fig. 7 that the intensity of the crystallization (*T*_c_) and melting (*T*_m_) transition peaks are decreasing and getting broader while changing the number of carbon atoms within α,ω-ALD from 6, 8 to 10, or 12. Moreover, for PDF-DLF and PDDF-DLF copolymers once the glass transition temperature is exceeded during the second heating step, the mobility of macromolecules is gained and further rearrangements take place, as shown by the cold crystallization peak (*T*_cc_). This indicates that the crystallization rate of those materials is slower at the tested time scale and that the crystalline phase in this case is less homogenous and crystallites with variable size distribution are formed. This is also in accordance with the conclusions drawn from XRD data that increasing number of carbon atoms within α,ω-ALD hinders crystallization due to the decreasing chain regularity. Of course, herein again, we also cannot neglect the fact, the differences in thermal transitions of PDF-DLF and PDDF-DLF copolyesters can be also influenced by higher values of averaged molecular weights where *M*_n_ and *M*_w_ are varying from 5700 to 20 600 g mol^−1^ and from 10 700 to 45 700 g mol^−1^, respectively. As was concluded before, it can also affect the crystallization behavior of those copolyesters by reducing the polymer chain mobility. However, similar studies were performed by Loos *et al.*^10^ in which they investigated the thermal properties of the 2,5-furandicarboxylic acid-based polyesters that consist of the furan units and α,ω-ALD units possessing different chain lengths (4, 6, 8, and 10 carbon atoms within diol unit). It was found that *T*_g_, *T*_m_, and *T*_c_ of 2,5-furandicarboxylic acid-based aliphatic polyesters showed a continuous decrease with an increasing number of carbon atoms within α,ω-ALD from 4 to 10, which is also in agreement with our observations.

To assess the potential application of furan-based copolyesters as environmentally friendly packaging materials for medical sector, we conducted oxygen transmission rate (OTR) measurements on 100µm-thick polymer films that were hot-pressed. Oxygen permeability assessment of packaging materials serves multiple purposes, including evaluating their barrier properties, assessing product shelf life and stability, and complying with regulatory requirements. In this study, our main focus was on measuring the oxygen transmission rate (OTR) to evaluate the suitability of furan-based copolyesters for the sterile barrier system, particularly within the context of medical packaging. While the excellent barrier properties of PEF or PBF materials are known for food packaging sector, we used long aliphatic chains of diols to strike a balance between these properties and the necessary transmission of sterilizing agents, which are essential for medical devices packaging. The emphasis on oxygen transmission rate in medical packaging primarily relates to the efficient ventilation of ethylene oxide after sterilization, ensuring the safety and integrity of packaged medical products. Therefore, understanding and optimizing the oxygen transmission properties of packaging materials are crucial in medical applications.

In our study, we conducted oxygen permeability assessments on three types of polymer films: POF-DLF, PDF-DLF, and PDDF-DLF. Unfortunately, we were unable to measure the OTR of the polymer film obtained from the PHF-DLF copolyester due to its brittleness. Table 5 displays the OTR values of the films, revealing an increasing trend as the number of carbon atoms within α,ω-ALD increased. The OTR values ranged from 410 to 641 cm^3^ m^−2^ 24 h. This increase can be attributed to a decrease in the crystalline phase content as longer chain lengths of α,ω-ALD were used. Consequently, oxygen molecules can more easily permeate through the amorphous phase, following a less tortuous path. Comparing the OTR values of furan-based copolyesters to materials commonly used in the packaging sector, we observe that the obtained values are comparable to those of HD-PE (Tyvek, the most popular materials for medical device packaging) or PBS. Furthermore, the tested materials demonstrate significantly lower oxygen transmission rates when compared to PBAT, PP, and LD-PE. These findings may suggest that the furan-based copolyesters exhibit promising oxygen permeability properties suitable for the medical packaging sector. The OTR values of these materials are comparable to those of certain commonly used materials, indicating their potential to allow for the necessary transmission of gases used for sterilization and subsequent ventilation after sterilization, while still ensuring the safety of the packaged medical products. The balance between barrier properties and the necessary transmission of sterilizing agents is a critical consideration in medical packaging applications, and the furan-based materials in our opinion demonstrate the potential in achieving this balance.

**Table 5:** Oxygen transmission rates of furan-based copolyesters and reference materials^a^

Copolymer	OTR [cm^3^ m^−2^ 24 h]	Sample thickness [µm]
POF-DLF	410 ± 29	107
PDF-DLF	489 ± 58	104
PDDF-DLF	641 ± 37	112
HD-PE (Tyvek)^b^	450–600	100
LD-PE^b^	≥2000	25
PP^b^	1800	40
PBAT^b^	1050	80
PBS^b^	400	70–80

a HD-PE (Tyvek) – high-density polyethylene, LD-PE – low-density polyethylene, PBAT – poly(butylene adipate terephthalate), PP – polypropylene, PBS – poly(butylene succinate).

b Data obtained from ref. 42–46.

In addition to the oxygen permeability assessment, we also conducted Dynamic Mechanical Thermal Analysis (DMTA) to evaluate the mechanical performance of the furan-based copolyesters (Fig. 9).

The DMTA measurements provided insights into the viscoelastic properties of the furan-based copolyesters. Below the glass transition temperatures (*T*_g_), the storage modulus remained constant, indicating a relatively rigid and solid-like behavior within this temperature range. However, when the glass transition temperature was reached, a noticeable decrease in the storage modulus values was observed. This transition represents a change in molecular mobility and is typically associated with a decrease in stiffness. It indicates that the material becomes more flexible, and exhibits increased molecular movement, which can affect its mechanical properties. As the temperature increased further, the storage modulus gradually decreased until reaching the melting temperature (*T*_m_). The results of the study revealed that the storage modulus (*E*′) was influenced by the chain length of α,ω-ALD, and averaged molecular masses of the copolymers, as well as by the different microstructure and blockiness, as deduced from discrepancies in the dispersity index (*Đ*) (3.0 for POF-DLF and 2.2 for PDF-DLF and PDDF-DLF). In particular, the copolyester with a lower molecular mass, POF-DLF, exhibited lower values of storage modulus compared to PDDF-DLF, which had higher averaged molecular masses. This relationship between molecular mass and mechanical properties highlights the importance of molecular structure in determining the stiffness and viscoelastic behavior of the materials. Overall, the values obtained from the DMTA analysis indicate that the furan-based copolyesters can be adjusted to meet specific requirements for packaging applications. By understanding the viscoelastic properties and the factors that influence the storage modulus, it becomes possible to tailor the material composition and molecular structure to achieve desired stiffness, flexibility, and other mechanical properties, however more studies are needed.

## 4. Conclusions

In this study, a new series of entirely bio-based copolymers containing poly(alkyl furanoate) as a hard segment and poly(dilinoleic furanoate) (DLF) as a soft segment with a 70–30 wt% ratio, respectively, were synthesized *via* an enzymatic process in diphenyl ether. The use of an immobilized form of enzyme allowed to perform the synthesis at a relatively high temperature (140 °C) enforced by the high melting temperatures of the furan-based copolyesters and poor solubility of the final products. The synthesis resulted in four different furan-based copolymers with high reaction yields (>89%).

The chemical structure of the copolymers was evaluated using ^1^H and ^13^C NMR spectroscopy, which confirmed that the copolyesters had the expected chemical structure. Additionally, the segmental composition of the copolyesters was calculated and found to be similar to theoretical values, indicating good control over the synthesis process. The content of hard segments in the copolyesters was also observed to increase with an increasing number of carbon atoms in the α,ω-ALD. This was likely due to the removal of diols with lower boiling points from the reaction mixture under the applied vacuum and high temperatures. Furthermore, the number averaged molecular weight of the copolyesters calculated from ^1^H NMR increased with an increasing number of carbon atoms in the α,ω-ALD.

SEC analysis revealed that using longer chain length α,ω-ALD in the polymerization process resulted in copolymers with higher molecular weights. This was mainly attributed to the increased enzymatic reactivity and selectivity of CAL-B, which is determined by the size and shape of its active site, enabling it to accommodate substrates of specific sizes and shapes, as visualized in the proposed mechanism. It was also correlated with decreased solubility of the resulting polyesters with a shorter chain length of α,ω-ALD. Additionally, the study revealed that the melting temperatures of the copolymers were influenced by the chain length of the α,ω-ALD used in the synthesis, with longer chain length resulting in lower melting temperatures. These findings underscore the importance of considering the properties of the starting materials in the enzymatic synthesis of bio-based copolymers.

X-ray diffraction analysis revealed that the furan-based copolyesters are semicrystalline materials and they crystallize into β-form crystals. The study found that the diol chain length within the hard segments had an effect on the crystalline structure of the copolyesters. As the chain length increased, the spacing between the planes in the atomic lattice (*d*) also increased. Additionally, the degree of crystallinity 
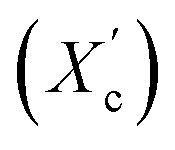
 decreased as the number of carbon atoms within α,ω-ALD increased. This is likely due to the decrease in chain regularity and greater capacity of rotational motions of polymer chains which may hinder the ability to form crystal structures.

The study also investigated the behavior of furan-based copolyesters during crystallization using holographic imaging and profiling. The results showed that the spherulite diameters fall within the range of 4–7 µm and the phase change between the crystalline and amorphous phase was smaller for certain copolymers, indicating a higher degree of crystallinity or more homogeneous distribution of the amorphous phase. The diol structure was also found to have a significant impact on the homogeneity of the crystallization process, with increasing *S*_a_ values as the number of atoms in the diol structure increases.

Finally, the combination of OTR measurements and DMTA analysis provided valuable insights into the suitability of the copolyester as medical packaging materials. The comparable OTR values to commonly used material in medical devices packaging such as Tyvek, and the ability to adjust material properties through α,ω-ALD selection highlight the potential of the copolyester for fulfilling specific requirements in medical packaging sector.

In summary, this research has successfully synthesized new bio-based copolymers and demonstrated the significant impact that the properties of starting materials have on the enzymatic synthesis process. The study sheds light on the relationship between diol chain length and the crystalline structure of the copolymers, providing valuable information for optimizing the properties of these materials for greener packaging materials in the medical sector. The use of monomers and enzymes derived from biomass in the synthesis of bio-based copolymers is a significant step towards resource-efficiency optimization.

Nevertheless, further improvements are needed with respect to the synthesis process and the selection of greener solvents to establish a fully sustainable platform for copolymer production. One promising solution to replace the relatively toxic diphenyl ether is the utilization of ionic liquids as a synthesis solvent.^47^ The replacement of chloroform with less toxic dimethoxyethane (DME) for polymers workup can be an environment friendly alternative, thus further studies are still needed in this matter.

## Author contributions

Martyna Sokołowska was responsible for methodology, investigation, validation, formal analysis, writing – original draft, and visualization. Jagoda Nowak-Grzebyta and Ewa Stachowska performed experiments on digital holographic microscope. Piotr Miądlicki and Beata Michalkiewicz performed XRD measurements. Magdalena Zdanowicz performed oxygen transmission rate measurements. Miroslawa El Fray was responsible for conceptualization, supervision, writing – review & editing, project administration and funds raising.

## Conflicts of interest

The authors declare that there are no conflicts to declare.

## Supplementary Material

RA-013-D3RA03885H-s001
