# Supplementary material for: Enzymatically catalyzed furan-based copolyesters containing dilinoleic diol as a building block
Source: RSC Adv. 2023 Jul 24;13(32):22234–49. doi: 10.1039/d3ra03885h (PMC10363961; doi:10.1039/d3ra03885h)
Supplement: RA-013-D3RA03885H-s001 [file RA-013-D3RA03885H-s001.pdf]

Electronic Supplementary Material (ESI) for RSC Advances.
This journal is © The Royal Society of Chemistry 2023

Supporting Information

Enzymatically catalyzed furan-based copolyesters containing dilinoleic diol as a building block

Martyna Sokołowska[a], Jagoda Nowak-Grzebyta[b], Ewa Stachowska[b], Piotr Miądlicki[c], Magdalena Zdanowicz[d], Beata Michalkiewicz[c], Miroslawa El Fray[a*]

[a] Department of Polymer and Biomaterials Science, West Pomeranian University of Technology, Szczecin, Faculty of Chemical Technology and Engineering, Al. Piastow 45, 71-311 Szczecin, Poland
[b] Poznan University of Technology, Faculty of Mechanical Engineering, ul. Piotrowo 3, 60-965 Poznan, Poland
[c] Engineering of Catalytic and Sorbent Materials Department, West Pomeranian University of Technology, Szczecin, Faculty of Chemical Technology and Engineering, Al. Piastow 45, 71-311 Szczecin, Poland
[d] Center of Bioimmobilisation and Innovative Packaging Materials, Faculty of Food Sciences, West Pomeranian University of Technology in Szczecin, ul. Janickiego 35, 71-270, Szczecin, Poland.
*mirfray@zut.edu.pl

Table of contents

Proton and Carbon Nuclear Magnetic Resonance (^{1}H and ^{13}C NMR) 2

Segmental composition and molecular mass calculation from ^{1}H NMR (Example for PHF-DLF) 6

The Fourier Transform Infrared Spectroscopy (ATR-FTIR) 8

Size Exclusion Chromatography (SEC) 10

Digital holographic microscope (DHM) 11

Differential Scanning Calorimetry (DSC) 12

Proton and Carbon Nuclear Magnetic Resonance (^1H and ^{13}C NMR)

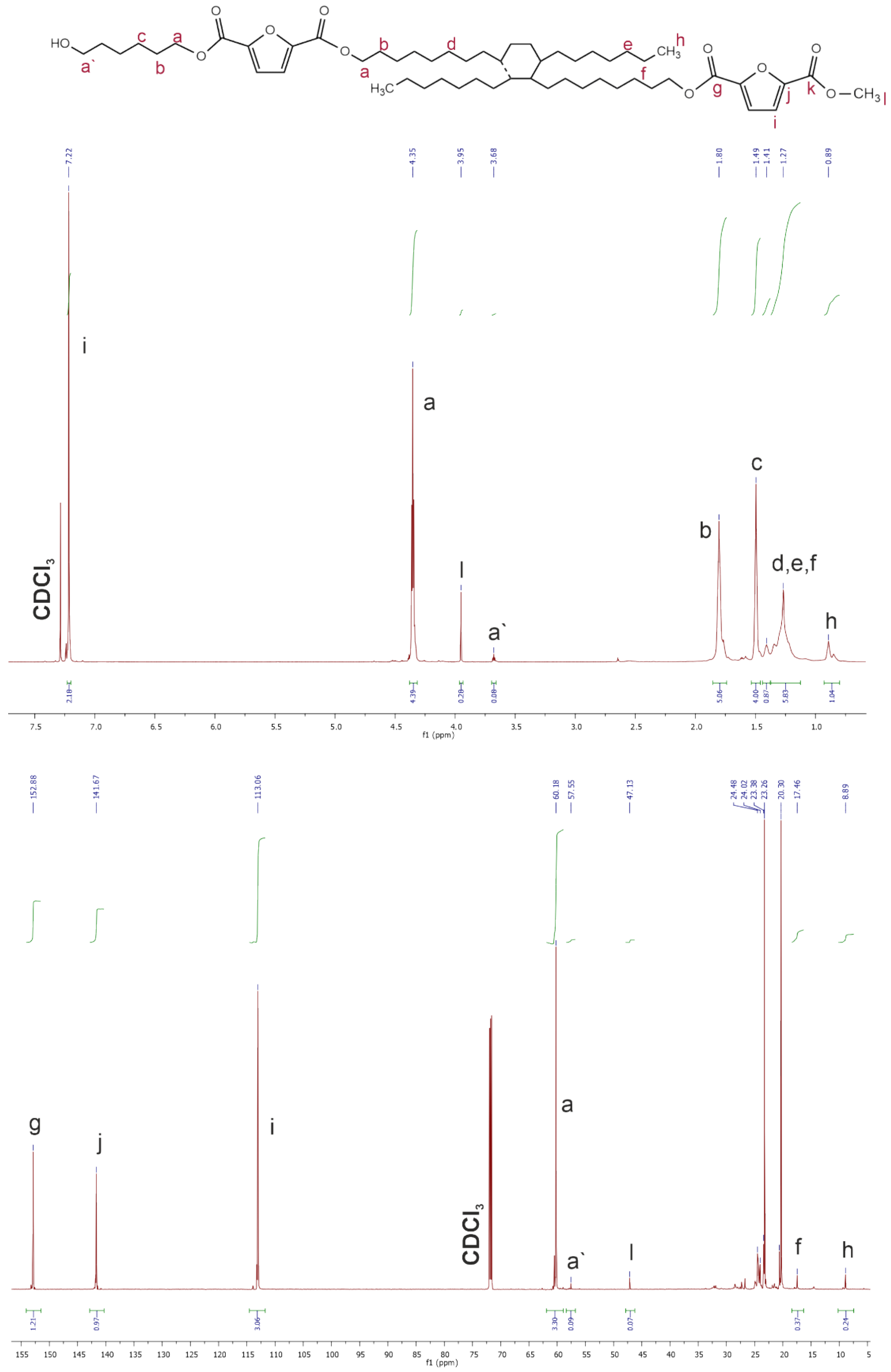

Figure S1. ^1H (up) and ^{13}C NMR (bottom) spectra of PHF-DLF copolyester

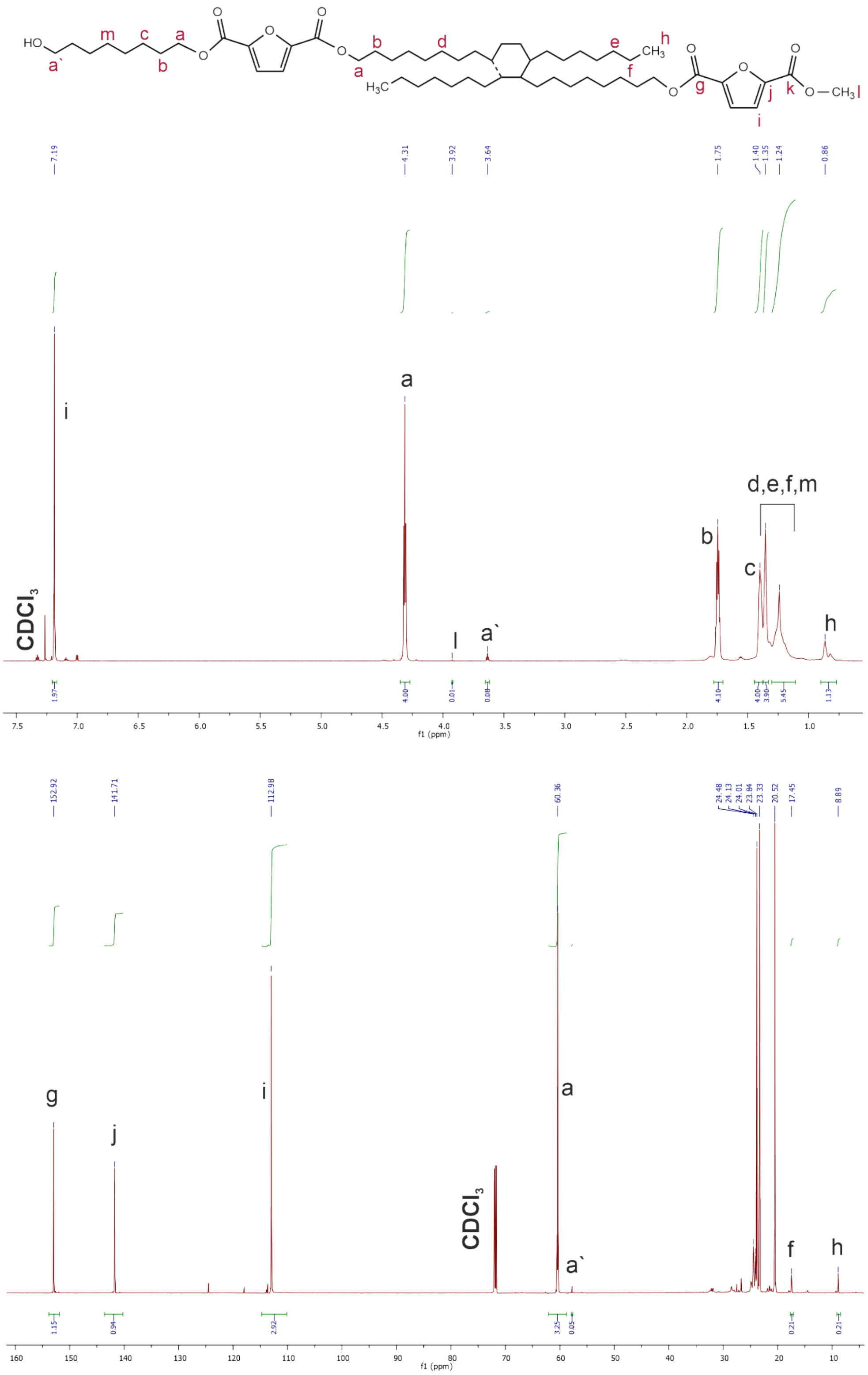

Figure S2. ^{1}H (up) and ^{13}C NMR (bottom) spectra of POF-DLF copolyester

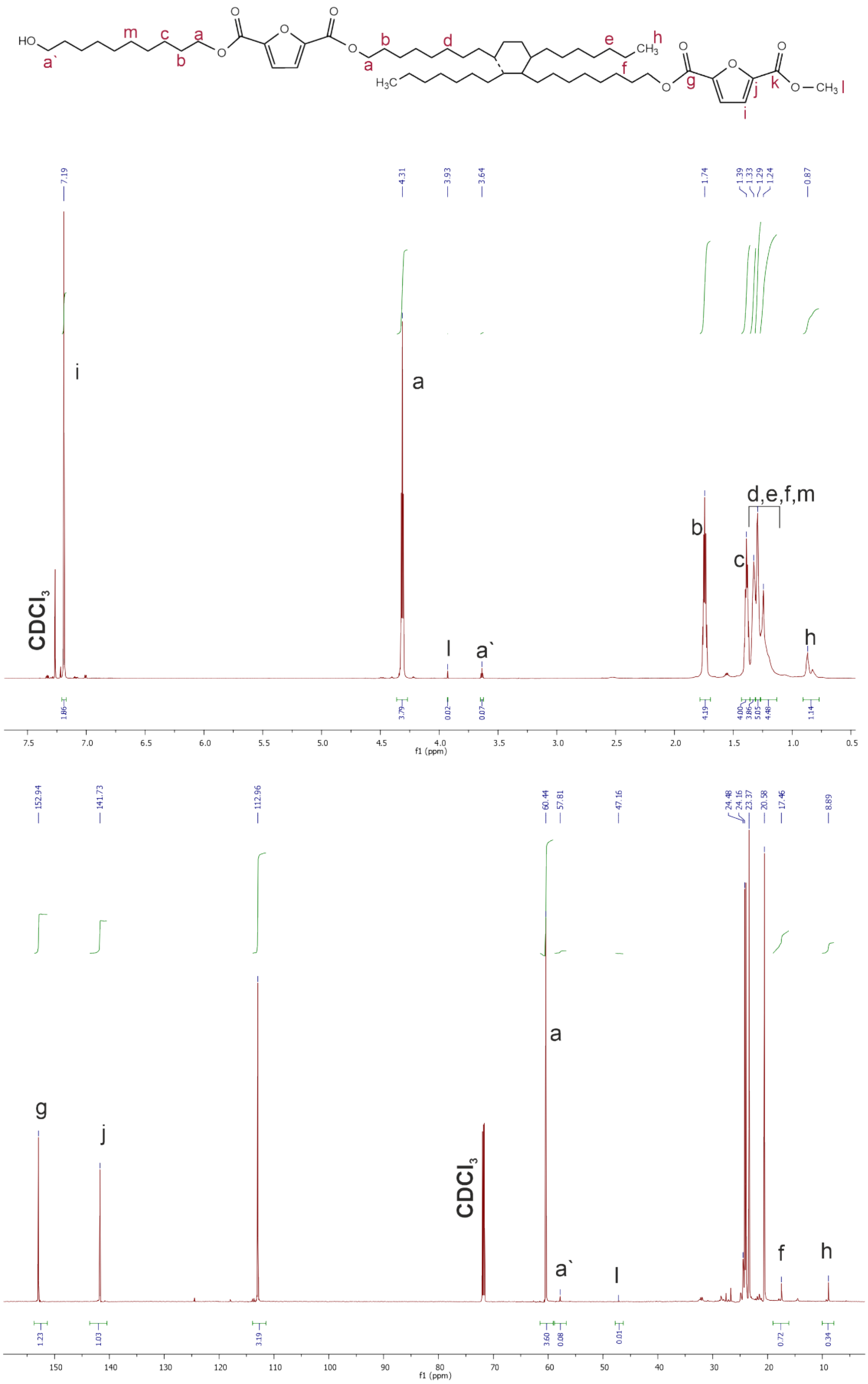

Figure S3. ^{1}H (up) and ^{13}C NMR (bottom) spectra of PDF-DLF copolyester

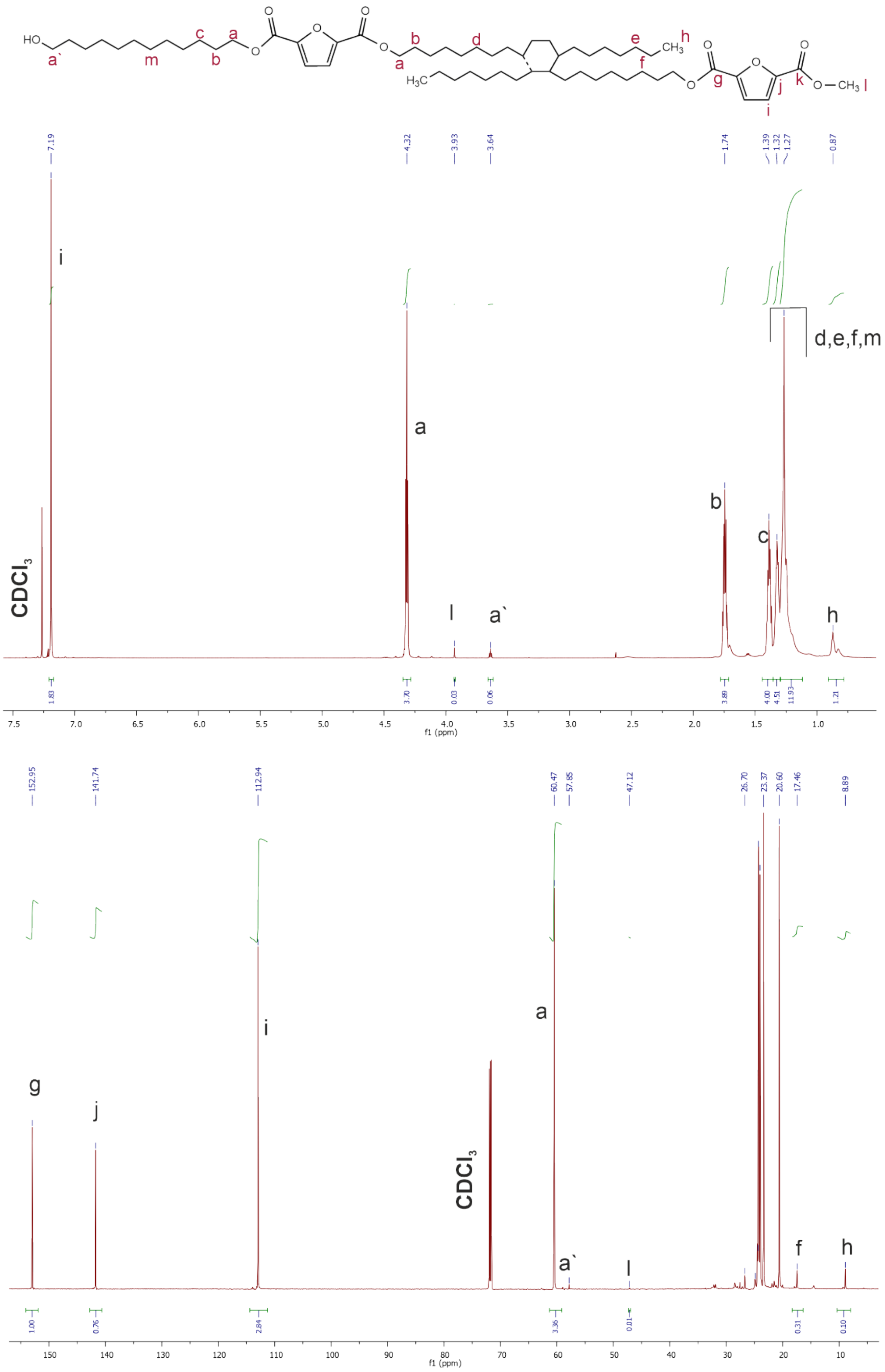

Figure S4 ^{1}H (up) and ^{13}C NMR (bottom) spectra of PDDF-DLF copolyester

Segmental composition and molecular mass calculation from ^{1}H NMR (Example for PHF-DLF)

Since the peak at 1.49 ppm (c) is related to four protons in the hexanediol (1,6-HDO) sequence (-CH_2-) of PHF segment and the peak at 0.89 ppm (h) is arising from six protons in the dilinoleic diol (DLD) sequence (-CH_3) of DLF segment, DP_h was computed using equation (1):

$$DP_h = \frac{\frac{I_{1,49}}{n_{1,49}}}{\frac{I_{0,89}}{n_{0,89}}} = \frac{\frac{4}{4}}{\frac{1.04}{6}} = 5.77 \qquad (1)$$

DP_h is a degree of polymerization of PHF hard segments, $I_{1,49}$ is the integral of signal at 1,49 ppm and $I_{0,89}$ is the integral of signal at 0,89 ppm. The weight percentage of hard segments ($\%W_h$) of PHF-DLF 70-30 copolyester was computed from DP_h using equation (2):

$$\%W_h = \frac{DP_h \cdot M_h}{DP_h \cdot M_h + M_s} \cdot 100\% = \frac{5.77 \cdot 238}{5.77 \cdot 238 + 662} \cdot 100\% = 67.47\ \% \qquad (2)$$

where $\%W_H$ is weight percentage of PHF hard segments, M_h is the molecular weight of hard segment (238 g/mol) and M_s is the molecular weight soft segment (662 g/mol). Likewise, using the signals of and dilinoleic diol, we can also compute the mole percent of each block using equations (3-4):

$$[\%Mol_h] = \frac{\frac{I_{1,49}}{n_{1.49}}}{\frac{I_{1,49}}{n_{1,49}} + \frac{I_{0,89}}{n_{0,89}}} \cdot 100\% = \frac{\frac{4}{4}}{\frac{4}{4} + \frac{1.04}{6}} \cdot 100\% = 85.23\ \% \qquad (3)$$

$$[\%Mol_s] = \frac{\frac{I_{0,89}}{n_{0,89}}}{\frac{I_{1,49}}{n_{1,49}} + \frac{I_{0,89}}{n_{0,89}}} \cdot 100\% = \frac{\frac{0.95}{6}}{\frac{4}{4} + \frac{0.99}{6}} \cdot 100\% = 14.77\ \% \qquad (4)$$

where %Mol_h and %Mol_s are mol percentage of hard and soft segments, respectively. $n_{1,49}$ and $n_{0,89}$ are number of protons in 1,6-HDO and DLD units.

Following the equations (5-6) we can also calculate the number of hard and soft segments (Num_h and Num_s, respectively) by comparing the HDO and DLD signals to that of the end groups, which are signals arising from hydroxyl end-groups of 1,6-HDO at 3.68 ppm (-C**H$_2$**OH) and the macromolecules can be expected to be capped by a 1,6-HDO on either end, so there will be two such end-groups (n_{end}).

$$[Num_h] = \frac{(I_{1,49} \cdot n_{3,68} \cdot n_{end})}{I_{3,68} \cdot n_{1,49}} = \frac{(4 \cdot 2 \cdot 2)}{0.08 \cdot 4} = 50.0 \quad (5)$$

$$[Num_s] = \frac{(I_{0,89} \cdot n_{3,65} \cdot n_{end})}{I_{3,65} \cdot n_{0,89}} = \frac{(1.04 \cdot 2 \cdot 2)}{0.08 \cdot 6} = 8.6 \quad (6)$$

Finally, by multiplying the number of each block times the molecular weight of each block and summing, we are able to calculate molecular weight of copolymer using equation (7):

$$[M_n] = Num_h \cdot M_h + Num_s \cdot M_s = 50.0 \cdot 238 + 8.6 \cdot 662 = 17\,593 \approx 17\,600 \quad (7)$$

Table S1. Table summarizing ^{1}H NMR integral values characteristic for signals arising from hard and soft segments, including corresponding chemical shifts. These values were used to calculate weight and mole percentages (Wt. % and Mol %), the number of hard and soft segments (Num h and Num s), as well as molecular weight (*M*n).

Reagent	M.W.	ppm	Hydrogens	Integration	H/species	Wt.	Wt. %	Mol %	Num h	Num s	*M*n
PHF	238.00	1.49	4.00	4.00	1.00	238.0	67.47	85.23	50.0	8.6	17 600
DLF	662.00	0.89	6.00	1.04	0.17	114.7	32.53	14.77			
End-group (C**H$_2$**-OH) from 1,6-HDO	-	3.68	4.00	0.08	0.02	-	-	-			
**Reagent**	**M.W.**	**ppm**	**Hydrogens**	**Integration**	**H/species**	**Wt.**	**Wt. %**	**Mol %**	**Num h**	**Num s**	***M*n**
POF	266.00	1.40	4.00	4.00	1.00	266.0	68.09	84.15	50.0	9.4	19 500
DLF	662.00	0.86	6.00	1.13	0.19	124.7	31.91	15.85			
End-group (C**H$_2$**-OH) from 1,8-ODO	-	3.64	4.00	0.08	0.02	-	-	-			
**Reagent**	**M.W.**	**ppm**	**Hydrogens**	**Integration**	**H/species**	**Wt.**	**Wt. %**	**Mol %**	**Num h**	**Num s**	***M*n**
PDF	294.00	1.39	4.00	4.00	1.00	294.0	70.04	84.03	57.0	10.9	23 900

DLF	662.00	0.87	6.00	1.14	0.19	125.8	29.96	15.97			
End-group (CH_2-OH) from 1,10-DDO	-	3.64	4.00	0.07	0.0175	-	-	-			

Reagent	M.W.	ppm	Hydrogens	Integration	H/species	Wt.	Wt. %	Mol %	Num h	Num s	*M*n
PDDF	322.00	1.39	4.00	4.00	1.00	322.0	70.69	83.22	66.7	20.2	34 800
DLF	662.00	0.87	6.00	1.21	0.20	133.5	29.31	16.78			
End-group (CH_2-OH) from 1,12-DDDO	-	3.64	4.00	0.06	0.015	-	-	-			

The Fourier Transform Infrared Spectroscopy (ATR-FTIR)

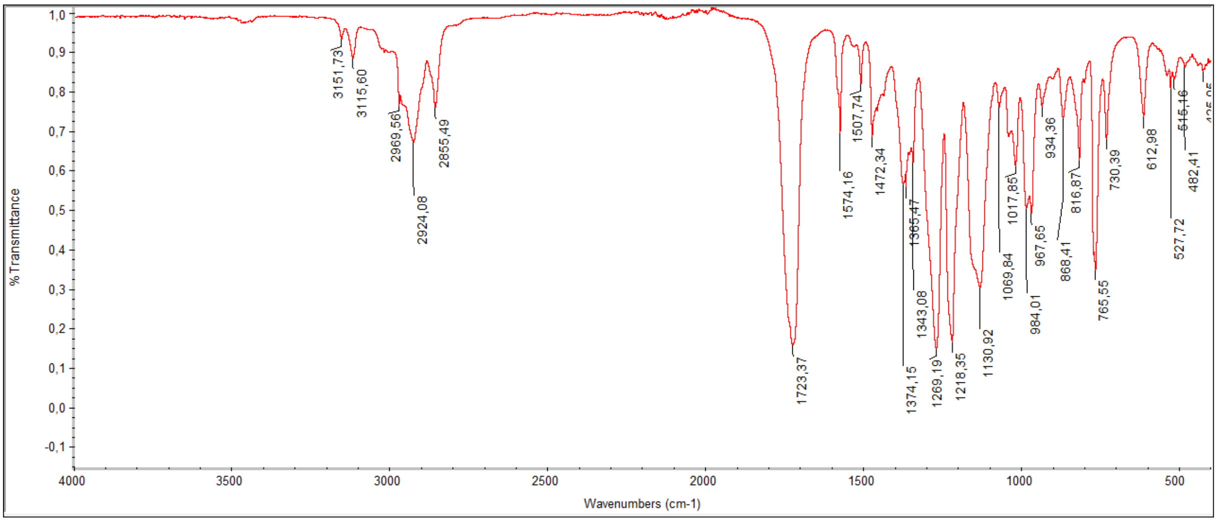

Figure S5. ATR-FTIR spectra of PHF-DLF copolyester.

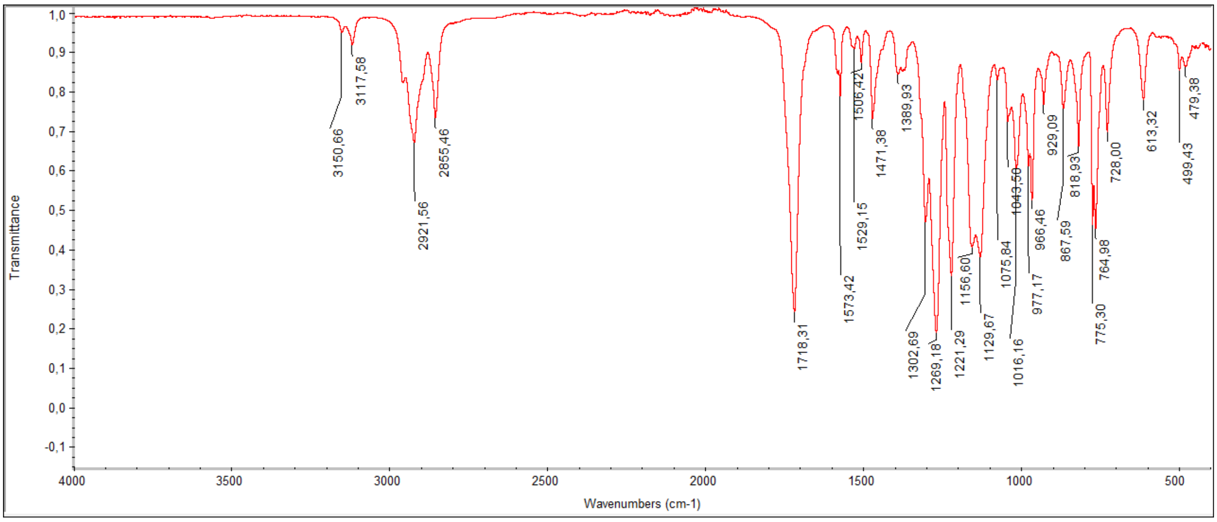

Figure S6. ATR-FTIR spectra of POF-DLF copolyester.

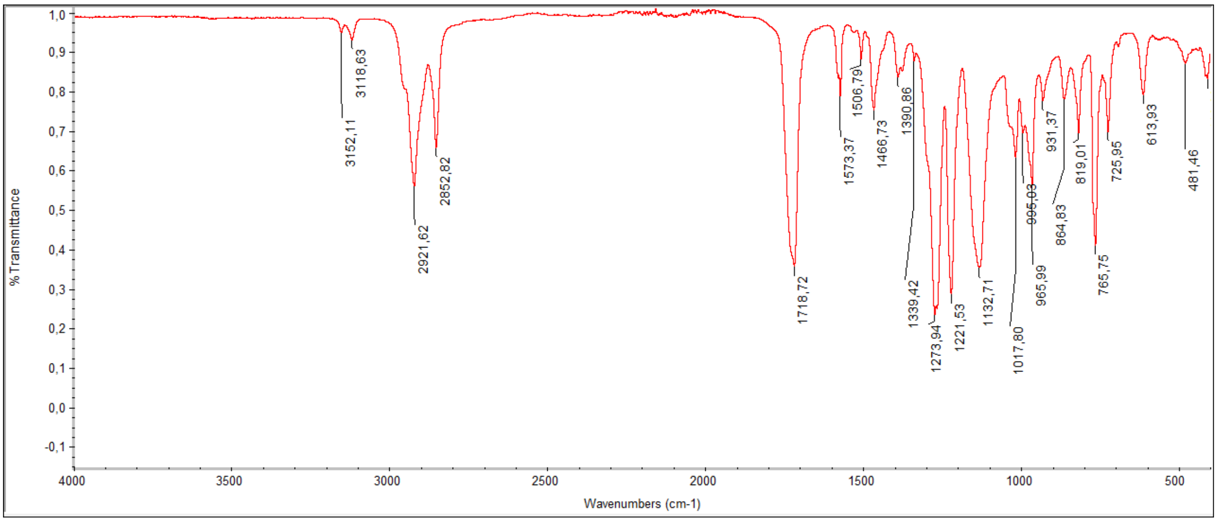

Figure S7. ATR-FTIR spectra of PDF-DLF copolyester.

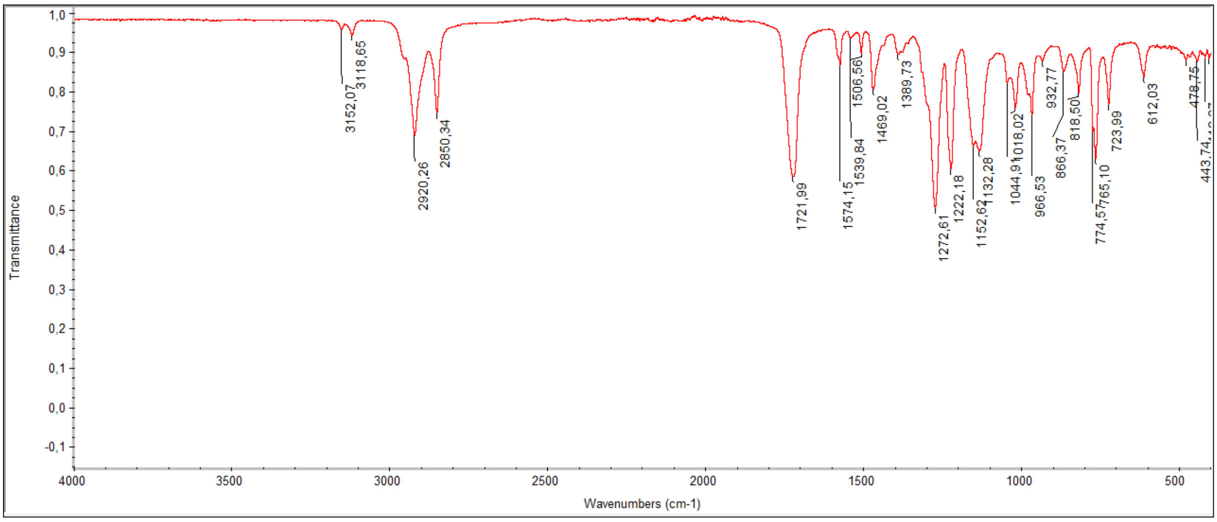

Figure S8. ATR-FTIR spectra of PDDF-DLF copolyester

Size Exclusion Chromatography (SEC)

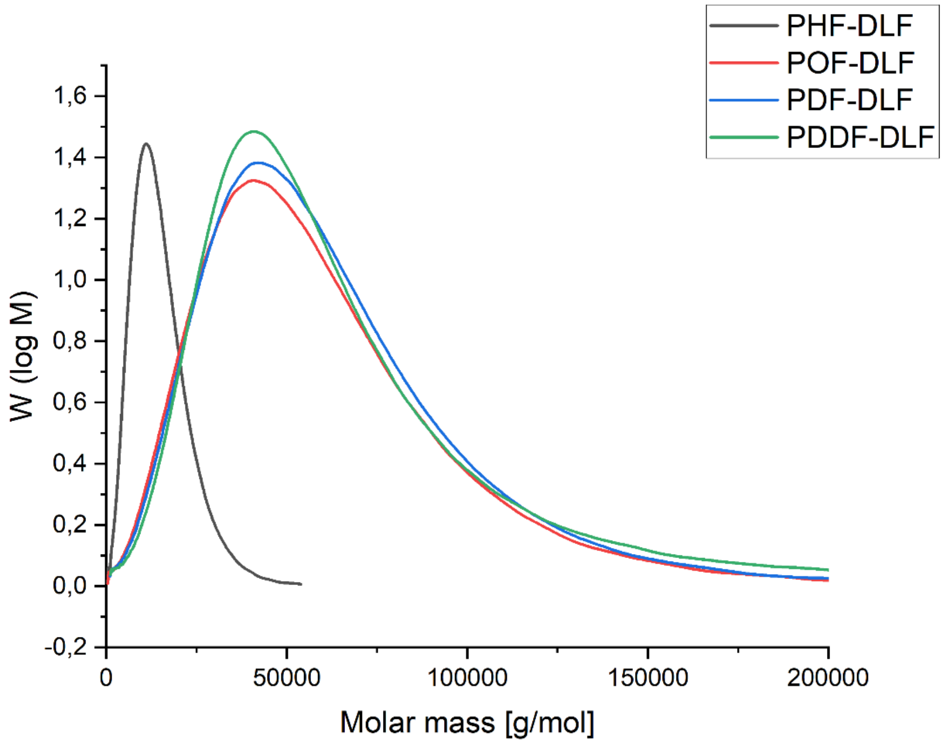

Figure S9. Overlay SEC curves presenting molar mass distribution.

Digital holographic microscope (DHM)

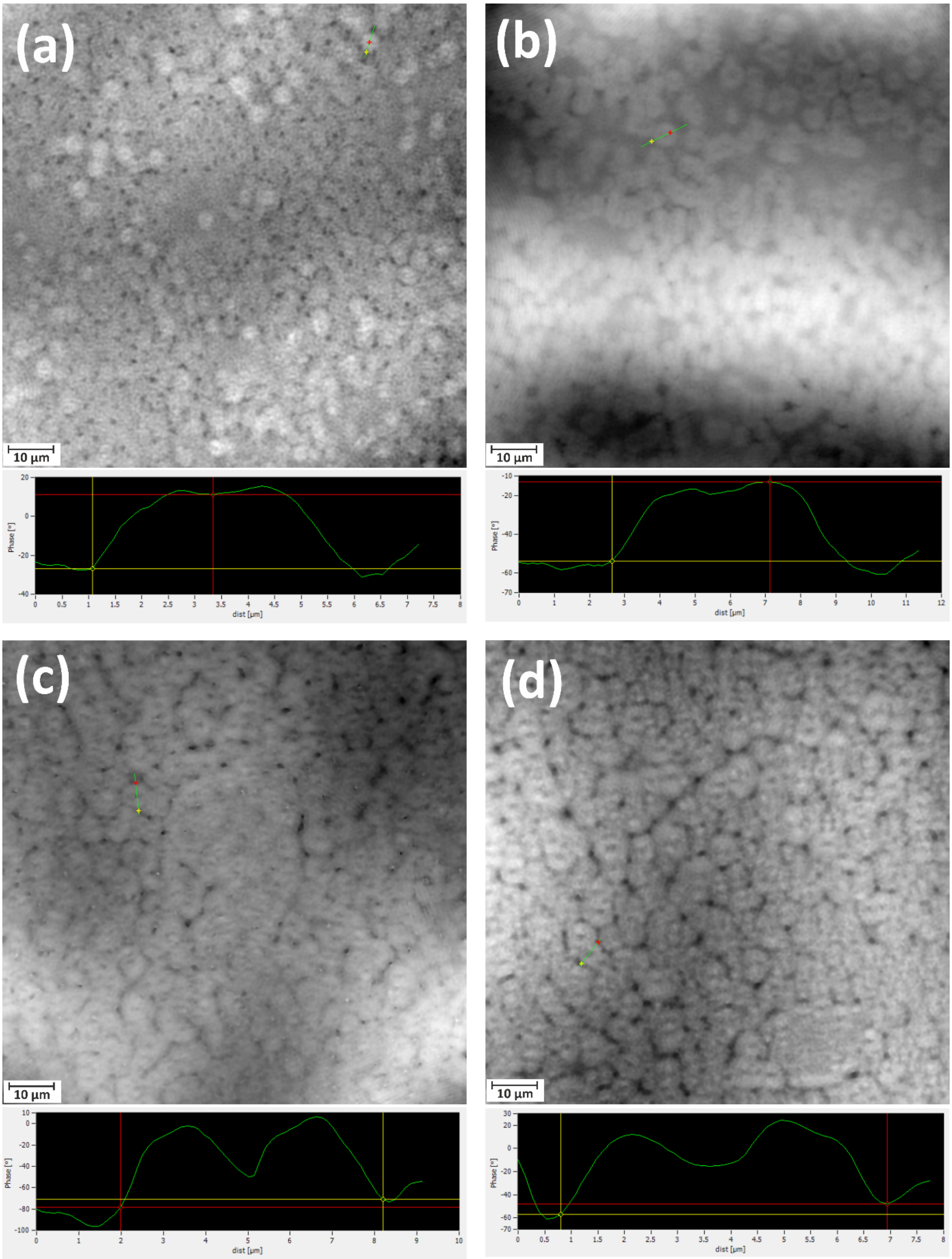

Figure S10. Holographic images and corresponding profiles of the phase changes along the green line recorded for a single spherulite crystallized from the melt for (a) PHF-DLF, (b) POF-DLF, (c) PDF-DLF, (d) PDDF-DLF. The DHM microscope magnification is 50x.

Differential Scanning Calorimetry (DSC)

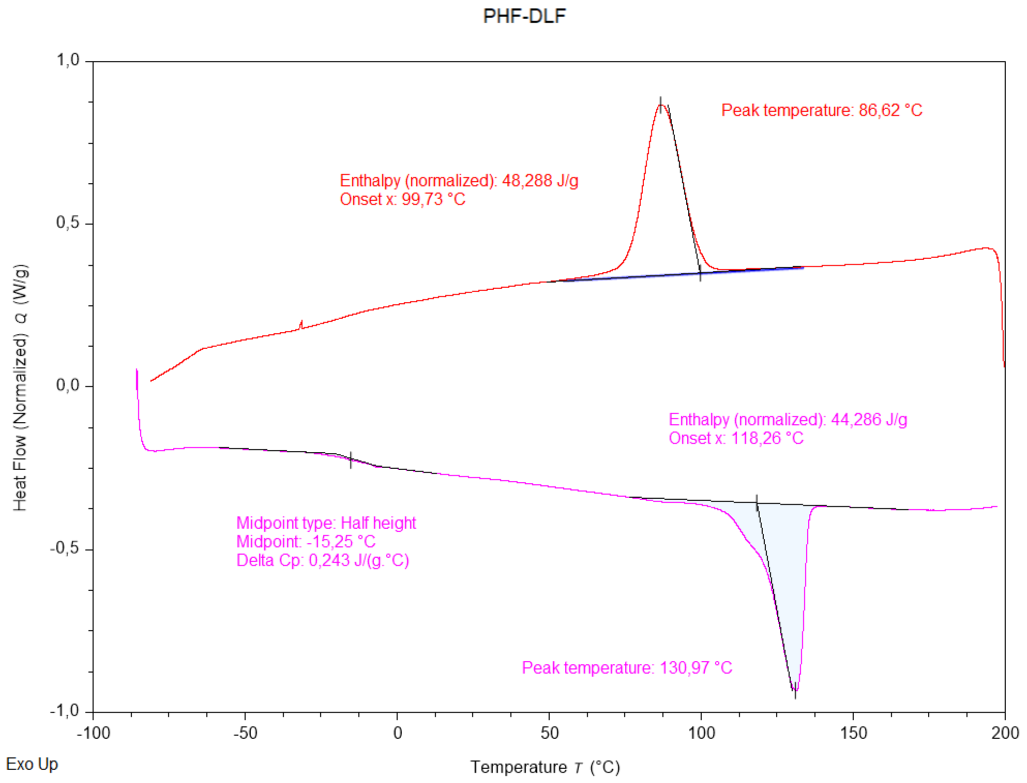

Figure S11. DSC II heating and cooling traces for PHF-DLF copolyester.

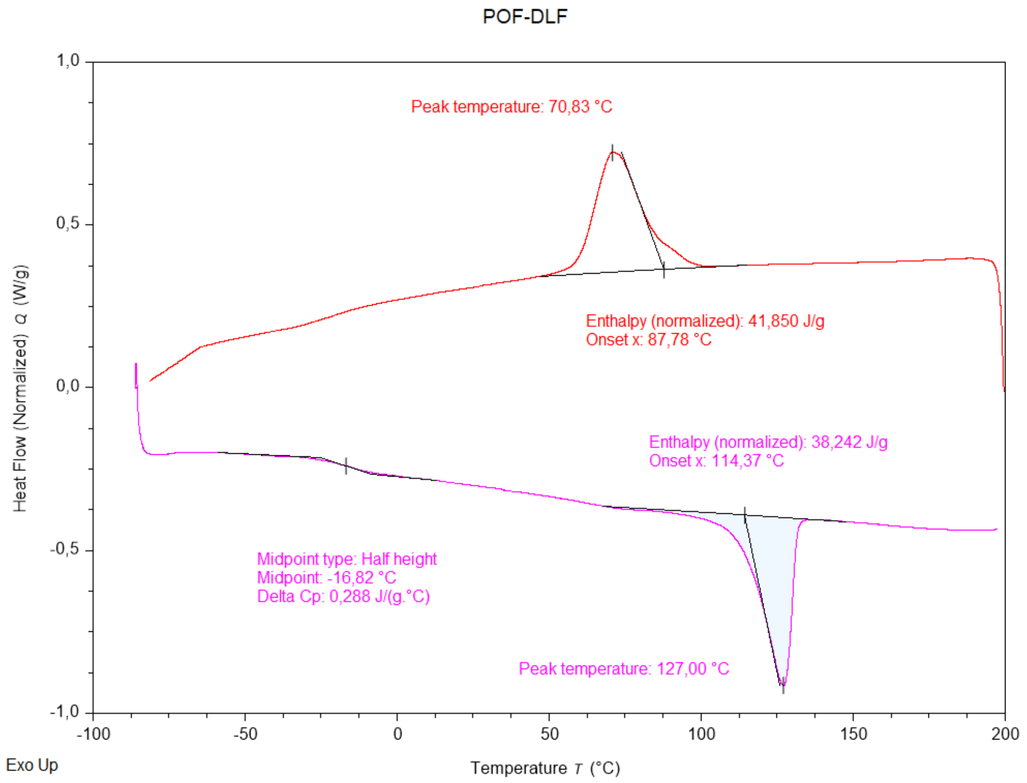

Figure S12. DSC II heating and cooling traces for POF-DLF copolyester.

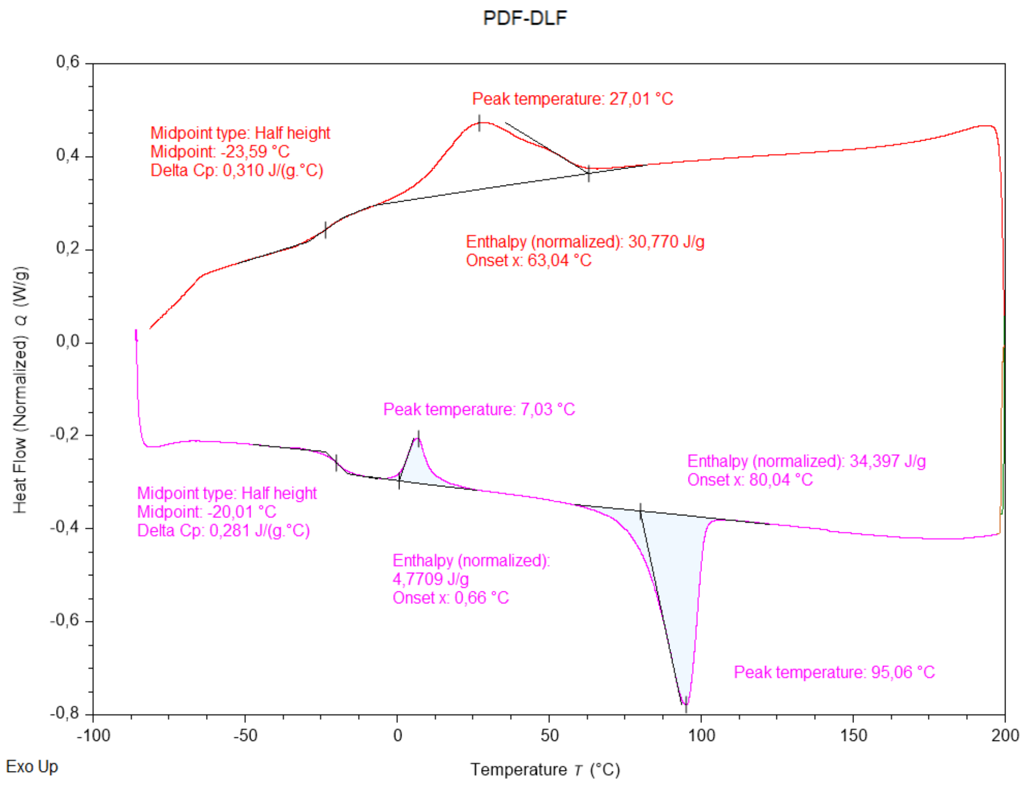

Figure S13. DSC II heating and cooling traces for PDF-DLF copolyester.

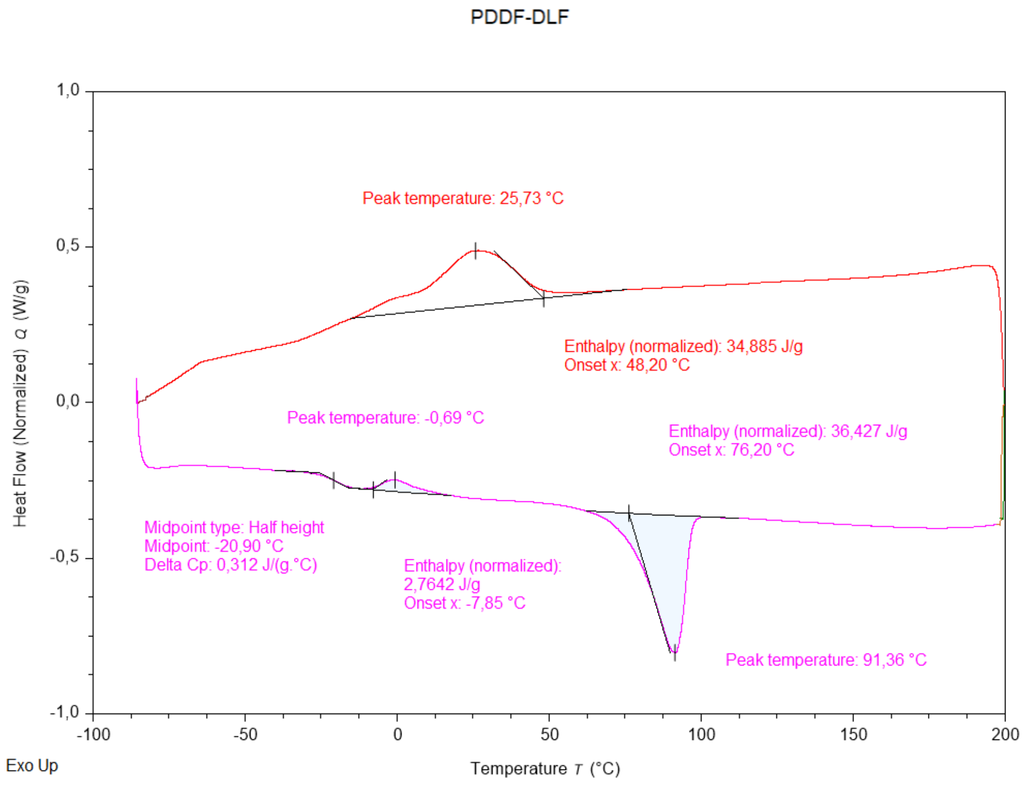

Figure S14. DSC II heating and cooling traces for PDDF-DLF copolyester.
